# Alkaline Ceramidase 3 Deficiency Results in Purkinje Cell Degeneration and Cerebellar Ataxia Due to Dyshomeostasis of Sphingolipids in the Brain

**DOI:** 10.1371/journal.pgen.1005591

**Published:** 2015-10-16

**Authors:** Kai Wang, Ruijuan Xu, Jennifer Schrandt, Prithvi Shah, Yong Z. Gong, Chet Preston, Louis Wang, Jae Kyo Yi, Chih-Li Lin, Wei Sun, Demetri D. Spyropoulos, Soyoung Rhee, Mingsong Li, Jie Zhou, Shaoyu Ge, Guofeng Zhang, Ashley J. Snider, Yusuf A. Hannun, Lina M. Obeid, Cungui Mao

**Affiliations:** 1 Department of Medicine, Stony Brook University, Stony Brook, New York, United States of America; 2 Stony Brook Cancer Center, Stony Brook, New York, United States of America; 3 Department of Hepatobiliary Surgery, Nanfang Hospital, Southern Medical University, Guangzhou, China; 4 Division of Rehabilitation Sciences, Department of Physical Therapy, School of Health Technology and Management, Stony Brook University, Stony Brook, New York, United States of America; 5 Department of Neurobiology and Behavior, Stony Brook University, Stony Brook, New York, United States of America; 6 Medical University of South Carolina, Charleston, South Carolina, United States of America; 7 Department of Gastroenterology, Nanfang Hospital, Southern Medical University, Guangzhou, China; 8 Biomedical Engineering and Physical Science Shared Resource, National Institute of Biomedical Imaging and Bioengineering, National Institute of Health, Bethesda, Maryland, United States of America; 9 Northport Veterans Affairs Medical Center, Northport, New York, United States of America; The Wellcome Trust Centre for Human Genetics, University of Oxford, UNITED KINGDOM

## Abstract

Dyshomeostasis of both ceramides and sphingosine-1-phosphate (S1P) in the brain has been implicated in aging-associated neurodegenerative disorders in humans. However, mechanisms that maintain the homeostasis of these bioactive sphingolipids in the brain remain unclear. Mouse alkaline ceramidase 3 (Acer3), which preferentially catalyzes the hydrolysis of C_18:1_-ceramide, a major unsaturated long-chain ceramide species in the brain, is upregulated with age in the mouse brain. Acer3 knockout causes an age-dependent accumulation of various ceramides and C_18:1_-monohexosylceramide and abolishes the age-related increase in the levels of sphingosine and S1P in the brain; thereby resulting in Purkinje cell degeneration in the cerebellum and deficits in motor coordination and balance. Our results indicate that Acer3 plays critically protective roles in controlling the homeostasis of various sphingolipids, including ceramides, sphingosine, S1P, and certain complex sphingolipids in the brain and protects Purkinje cells from premature degeneration.

## Author Summary

Bioactive sphingolipids, such as ceramides and sphingosine-1-phosphates, have been implicated in neurodegenerative diseases. However, it remains unclear as to how the homeostasis of these bioactive lipids is sustained. Alkaline ceramidase 3 (ACER3) catalyzes the hydrolysis of saturated long-chain ceramides (C_18:1_-ceramide and C_20:1_-ceramide) to generate sphingosine (SPH), which is phosphorylated to form sphingosine-1-phosphate (S1P). In this study we found that Acer3 is upregulated with age in the mouse brain and blocking Acer3 upregulation elevates the levels of ceramides while reducing S1P levels in the brain, thereby resulting in Purkinje cell loss and cerebellar ataxia. This study not only offers novel insights into the role for the homeostasis of ceramides and their metabolites in regulating normal aging of the cerebellum, but also provides a useful genetic tool to dissect the mechanism by which an aberrant accumulation of ceramides results in age-related neurological disorders.

## Introduction

Ceramides consisting of a sphingoid base moiety and an amide-linked acyl chain are the precursors of all complex sphingolipids that are essential for brain development, maturation, and proper functioning in mammals [1,2]. Ceramides are also essential precursors of sphingosine-1-phosphate (S1P), a bioactive lipid implicated in survival of neural stem cells and neurons [3,4]. On the other hand, an aberrant accumulation of ceramides in the brain has been implicated in various aging-related neurodegenerative diseases [5,6], likely due to their signaling roles in cellular stress responses such as apoptosis [7] and senescence [8]. These observations suggest that the homeostasis of ceramides must be fine-tuned in the brain during normal aging process, else pathological consequences may result. However, how this is accomplished remains unclear; although it is generally believed that cellular or tissue levels of ceramides are determined by a balance between their synthesis and utilization or degradation.

Ceramides are generated through multiple metabolic pathways in mammals [9]. They are formed from dihydroceramides [10,11], which in turn are synthesized *de novo* from dihydrosphingosine and fatty acyl-CoAs through the action of (dihydro)ceramide synthases encoded by 6 distinct genes (CERS1-6) [12–17]. Ceramides can also be directly synthesized from sphingosine (SPH) and acyl-CoAs by the action of the CERS through a salvage pathway [18,19]. Following their synthesis by these two pathways in the endoplasmic reticulum (ER) [20,21], ceramides are transported to the Golgi complex [22,23], where they are incorporated into complex sphingolipids [9], which can be converted back to ceramides either on the plasma membrane [24] or in lysosomes [25].

Once generated, ceramides are then hydrolyzed to SPH and fatty acids by the action of ceramidases encoded by five distinct ceramidase genes, including the acid ceramidase (*ASAH1*) [26], neutral ceramidase (*ASAH2*) [27], alkaline ceramidase 1 (*ACER1*) [28], alkaline ceramidase 2 (*ACER2*) [29], and alkaline ceramidase 3 (*ACER3*) [30]. These ceramidases have distinct cellular localizations, substrate specificities, and tissue distributions [31]. ASAH1, a lysosomal ceramidase, is ubiquitously expressed and preferentially catalyzes the hydrolysis of C_12-14_-ceramides, so called medium-chain ceramides [32]. ASAH2 is localized to mitochondria and the plasma membrane in a membrane-bound form [27] or it is secreted from cells after its membrane-anchoring domain is cleaved [33]. ASAH2, which is expressed in different tissues [27], has been shown to catalyze the hydrolysis of various ceramides ranging from long-chain (C_16-20_) (LCCs) to very long-chain (≥C_22_) ceramides (VLCCs) *in vitro* [34]. ACER1, an ER ceramidase that is predominantly expressed in the skin [28], uses VLCCs as substrates [28]. ACER2, a Golgi ceramidase that is ubiquitously expressed at low levels [29], has broad substrate specificity [35]. ACER3, which is localized to both the ER and Golgi complex and is highly expressed in various tissues, was the first mammalian alkaline ceramidase to be identified by our group [30]. Initially we found that ACER3 prefers a synthetic fluorescent phytoceramide (NBD-C_12_-phytoceramide) over fluorescent ceramide (NBD-C_12_-ceramide) or dihydroceramide (NBD-C_12_-dihydroceramide) as a substrate [30]. Later, we discovered that ACER3 catalyzes the hydrolysis of ceramides, dihydroceramides, and phytoceramides carrying an unsaturated long acyl chain (C_18:1_ or C_20:1_) with similar efficiency [36]

Increasing studies suggest that ceramidases may play a key role in regulating the homeostasis of both ceramides and S1P in cells and/or tissues by controlling the hydrolysis of ceramides and the generation of SPH, the immediate precursor of S1P [29,37–39]. Genetic studies of human diseases and knockout mouse models are beginning to shed light on the distinct roles of ceramidases. A genetic deficiency in ASAH1 causes a massive accumulation of ceramides in various tissues including the brain, leading to the lysosomal storage disease called Farber’s disease [32,40]. A mouse model of Farber’s disease also exhibits accumulation of ceramides in different tissues including the brain and displays various pathological phenotypes including neuronal degeneration [41]. Asah2 knockout does not alter the brain levels of ceramides or SPH [37], suggesting a minimal role for this enzyme in the homeostasis of ceramides in the brain. However, the role for the alkaline ceramidases (Acer1-3) in regulating the homeostasis of ceramides and S1P in the brain remains unclear although their activities have been shown to be increased with age in the mouse brain [42].

In this study, we demonstrate that Acer3, the most abundant member in the alkaline ceramidase family, is upregulated with age in the mouse brain; plays important roles in sustaining the homeostasis of ceramides and their metabolites SPH and S1P in the aging brain; and protects PCs from premature degeneration and thereby cerebellar ataxia.

## Results

### Acer3 is upregulated with age in the mouse brain

**Fig 1:**
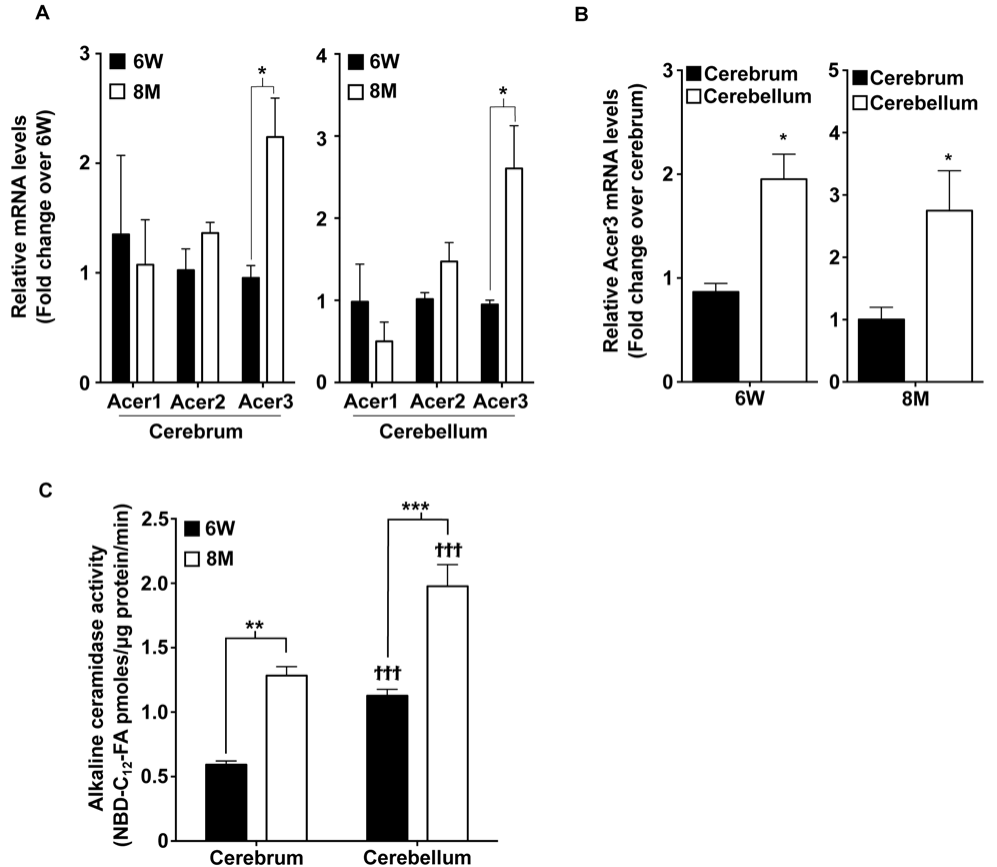
Acer3 is upregulated with age in the mouse brain. **A**. mRNA levels of the alkaline ceramidases Acer1, Acer2, and Acer3 in the cerebra and cerebella of young [6 weeks (6W) of age] versus middle-aged mice [8 months (8M) of age] Note that the mRNA levels of Acer3 but not Acer1 or Acer2 were increased in mice at 8M of age compared to at 6W. **B**. Acer3 mRNA levels in the cerebellum versus the cerebrum of 6W or 8M-old mice. Note that Acer3 mRNA levels were higher in the cerebellum than in the cerebrum in the same mouse at either 6W or 8M of age. **C**. Alkaline ceramidase activity on NBD-C_12_-PHC in the brains of young (6W) versus middle-aged mice (8M). Note that the alkaline ceramidase activity was higher in both cerebrum and cerebellum in 8M-old-mice compared to 6W-old mice and that the alkaline ceramidase activity was higher in the cerebellum than in the cerebrum in mice at either age. Data in A, B, and C represent mean values ± SD, n = 3.

It has been shown that alkaline ceramidase activity increases with age in the mouse brain [42]. We hypothesized that this ceramidase activity increase may be important in preventing an accumulation of ceramides in the mature brain by catalyzing the hydrolysis of ceramides. To test this possibility, we determined whether inhibiting the increase in ceramidase activity would lead to an aberrant accumulation of ceramides in the brain and consequent neurological disorder. To achieve this goal, we first determined which alkaline ceramidase(s) is upregulated with age in mouse brain. With quantitative real-time polymerase chain reaction **(**qPCR), we demonstrated that the mRNA levels of Acer3 but not Acer1 or Acer2 were increased in both cerebrum and cerebellum of C57BL6/J wild-type (WT) mice at 8 months of age compared to mice at 6 weeks of age (Fig 1A). We also found that Acer3 mRNA levels were higher in the cerebellum than in the cerebrum (Fig 1B). To determine if the increase in Acer3 mRNA levels results in an increase in its enzymatic activity, we measured alkaline ceramidase activity on NBD-*ribo-*C_12_-NBD-phytoceramide (NBD-C_12_-PHC), a synthetic substrate specific for the human ACER3 [30] and presumably for the mouse Acer3 as well. Our results showed that alkaline ceramidase activity toward NBD-C_12_-PHC was increased in both the cerebrum and cerebellum in 8-month-old WT mice compared to 6-week-old WT mice (Fig 1C), suggesting that Acer3 activity is indeed increased with age in the mouse brain. Correlating with Acer3 mRNA levels, we found that Acer3 activity was higher in the cerebellum than in the cerebrum (Fig 1C). These results suggest that Acer3 is upregulated with age in both the cerebrum and cerebellum.

### Acer3 knockout does not cause a major defect in mouse development

**Fig 2:**
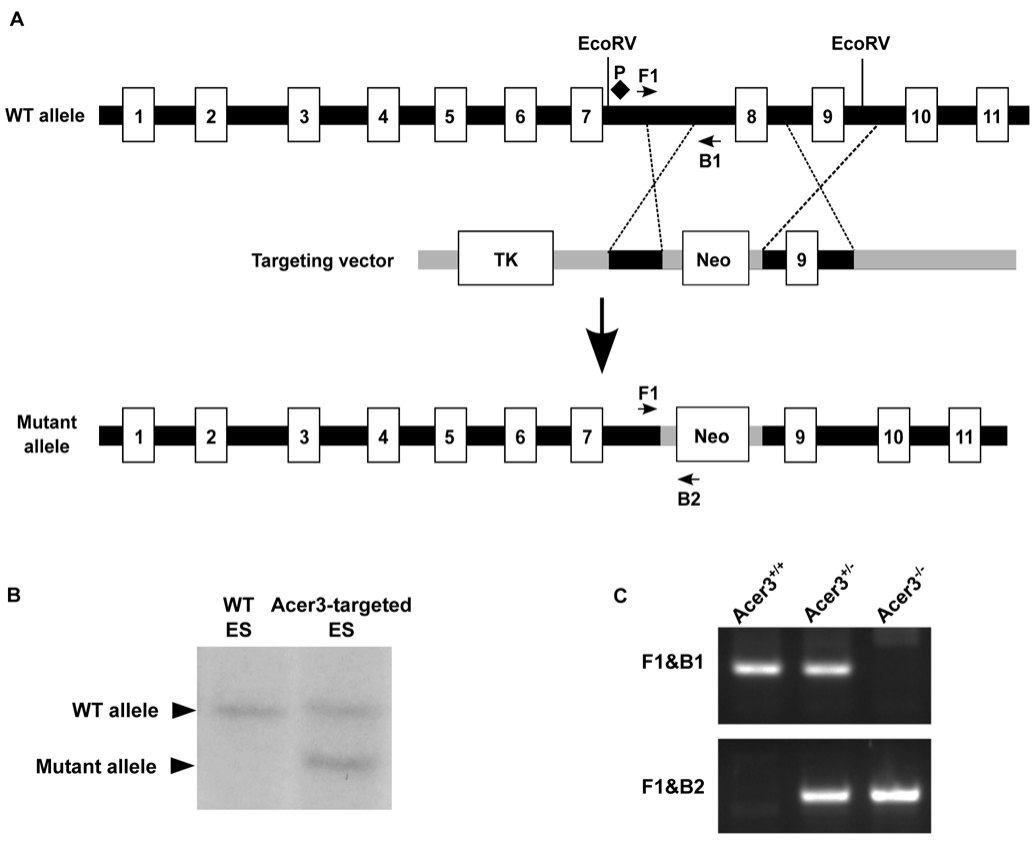
Generation of Acer3 null mouse. **A**. Acer3 targeting strategy. The Acer3 gene consists of 11 exons (empty rectangles with the numerals inside). Exon 8 of the Acer3 gene was replaced by the *Neo* resistant gene cassette upon homologous recombination. **B**. Southern blot analyses of WT ES cells or ES cells from an Acer3-targeted ES clone. Genomic DNA was digested with EcoRV, resolved on a 0.8% agarose gel, transferred to a nitrocellulose membrane, which was labeled with a radioactive probe (P) corresponding to the region upstream of Exon 8 as shown in Panel A. **C**. PCR-based genotyping of Acer3^+/+^, Acer3^+/-^, and Acer3^-/-^ mice. DNA was isolated from mouse tail biopsies and subjected to PCR analyses using the PCR primer pairs (F1 and B1 or F1 and B2) as shown in Panel A. The image in C represents the PCR product patterns of the three genotypes, Acer3^+/+^, Acer3^+/-^, and Acer3^-/-^.

**Fig 3:**
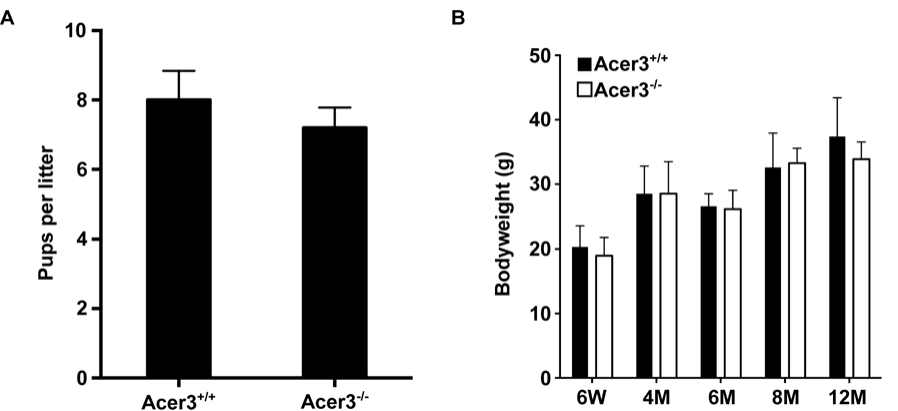
Acer3 knockout does not have any major defect in mouse development and fertility. **A**. The average litter size of Acer3^+/+^ or Acer3^-/-^ interbreeding mice (n = 4 litters per breeding pair) from 4 pairs of interbreeding mice. **B**. The average body weight of Acer3^+/+^ or Acer3^-/-^ mice at 6 weeks (6W), 4 months (4M), 6 months (6M), 8 months (8M), and 12 months (12M) of age, n = 5–10 per age group. Data in A and B represent mean values ± SD.

To elucidate the physiological function of age-dependent upregulation of Acer3 in the brain, we generated a mouse model deficient in Acer3 as described in Materials and Methods (Fig 2). Interbreeding of mice heterozygous for an *Acer3* null allele produced WT (Acer3^+/+^), heterozygous (Acer3^+/-^), and homozygous (Acer3^-/-^) offspring at a Mendelian ratio (Acer3^+/+^: Acer3^+/-^: Acer3^-/-^; 151:319:143), suggesting the absence of significant embryonic lethality in Acer3 knockout mice. Intercrossing of Acer3^-/-^ mice produced similar offspring numbers as with the intercrossing of Acer3^+/+^ mice (Fig 3A), suggesting that both male and female Acer3^-/-^ mice have normal fertility. There was no significant difference in body weight between Acer3^+/+^ and Acer3^-/-^ mice when measured at 6 weeks, 4, 6, 8, or 12 months of age (Fig 3B). The macroscopic and microscopic analyses found no abnormalities in the anatomy of major organs in young mice. These results suggest that Acer3 knockout does not appear to cause any major defect in mouse development at least prior to middle age.

### Acer3 knockout decreases alkaline ceramidase activity on unsaturated long-chain ceramides (ULCCs) in the brain

**Fig 4:**
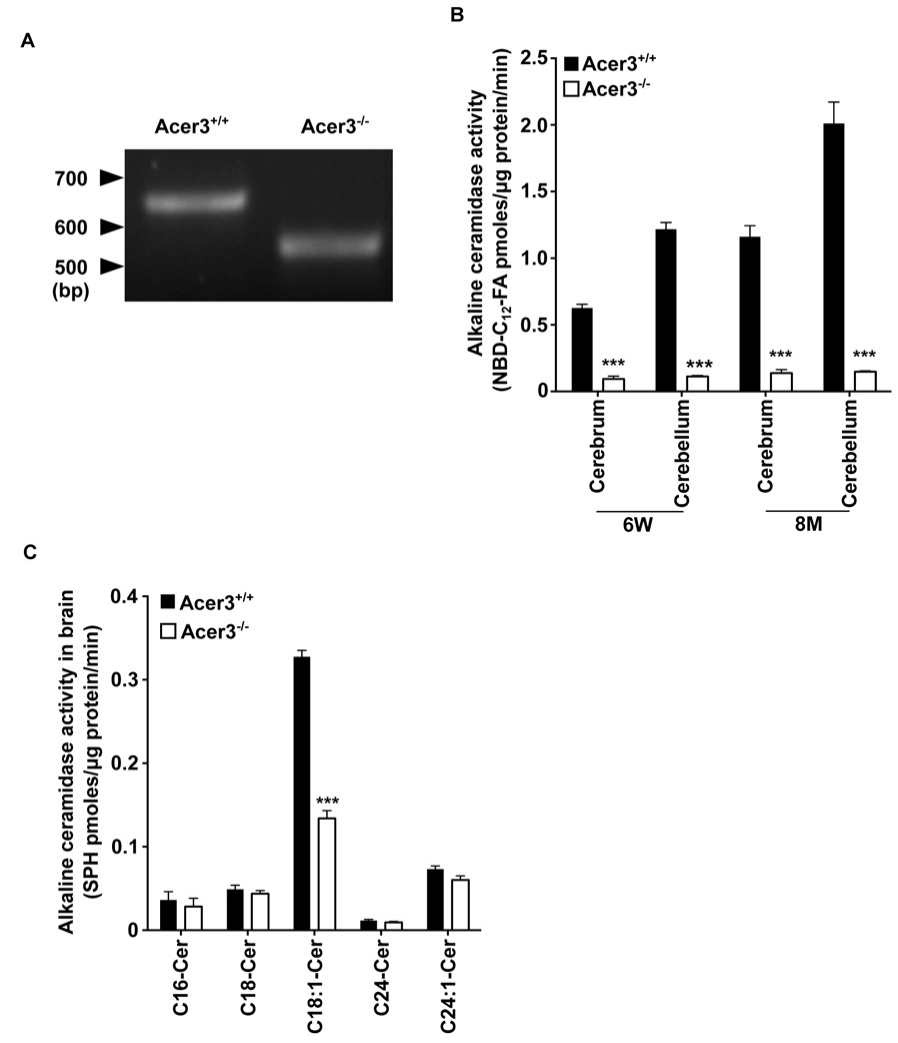
Acer3 knockout decreases alkaline ceramidase activity on ULCC in the brain. **A**. The transcription of a truncated coding sequence in a representative Acer3 knockout mouse. RNAs were isolated from the brains of Acer3^+/+^ or Acer3^-/-^ mice and subjected to RT-PCR using a pair of primers encompassing the start codon and stop codon, respectively, of the Acer3 gene. Note that the Acer3^-/-^ mouse has a smaller ORF of the Acer3 gene than an age-matched Acer3^+/+^ mouse. **B**. Reduction of alkaline ceramidase activity on NBD-C_12_-PHC in Acer3^-/-^ mice. Note that Acer3^-/-^ mice at either 6W or 8M of age show significant declines in ceramidase activity in both the cerebellar and cerebral brains compared to their WT littermates. **C**. Reduction of alkaline ceramidase activity on C_18:1_-ceramide in the whole brains of Acer3 knockout mice. Note that the brain alkaline ceramidase activity on this ceramide was substantially decreased in Acer3^-/-^ mice compared to Acer3^+/+^ mice. Image in A represents result from 3 pairs of mice. Data in B and C represent mean values ± SD, n = 3.

To confirm that the deletion of exon 8 of the Acer3 gene indeed results in a truncated coding sequence of Acer3, we amplified the coding sequence from cDNAs that are transcribed from RNAs from brain tissues of Acer3^+/+^ or Acer3^-/-^ mice. With reverse transcription PCR (RT-PCR), we demonstrated that the coding sequence of Acer3 in Acer3^-/-^ mice is shorter in length than that in Acer3^+/+^ mice (Fig 4A), suggesting that a truncated coding sequence is transcribed in Acer3^-/-^ mice.

To confirm that deleting exon 8 of the Acer3 gene inactivates the catalytic function of Acer3, we compared alkaline ceramidase activity on NBD-C_12_-PHC in the brain, liver, and lung tissues from Acer3^-/-^ and Acer3^+/+^ mice. We found that Acer3^+/+^ mice had much higher alkaline ceramidase activity on NBD-C_12_-PHC in these tissues than Acer3^-/-^ mice at either 6 weeks of age (Fig 4B and S1A Fig) or 8 months of age (Fig 4B) although this alkaline ceramidase activity was not totally abolished in some Acer3^-/-^ tissues. There are two possible explanations for these results: 1) the catalytic activity of Acer3 is abolished by deleting exon 8 and the residue alkaline ceramidase activity on NBD-C_12_-PHC comes from another ceramidase(s) with a minor activity on NBD-C_12_-PHC; and 2) the catalytic activity of Acer3 is markedly reduced but not abolished by the deletion of exon 8. These results also confirm that NBD-C_12_-PHC is a substrate highly specific for Acer3 and that the alkaline ceramidase activity on NBD-C_12_-PHC correlates with Acer3 expression levels in tissues.

The inactivation of the Acer3 gene was further confirmed by performing alkaline activity assays using regular ceramides carrying various acyl chains as substrates. The results showed that Acer3 knockout markedly decreased alkaline ceramidase activity on C_18:1_-ceramide, but not C_16:0_, C_18:0_, C_24:1_, or C_24:0_-ceramide in brain (Fig 4C), liver, or lung tissues (S1B Fig), further confirming that the deletion of exon 8 markedly reduces or inactivate the catalytic activity of Acer3. These results also suggest that, similar to its human counterpart ACER3, the mouse Acer3 also prefers ULCCs as substrates.

### Acer3 knockout leads to dyshomeostasis of ceramides and their sphingolipid derivatives in the aging brain

**Fig 5:**
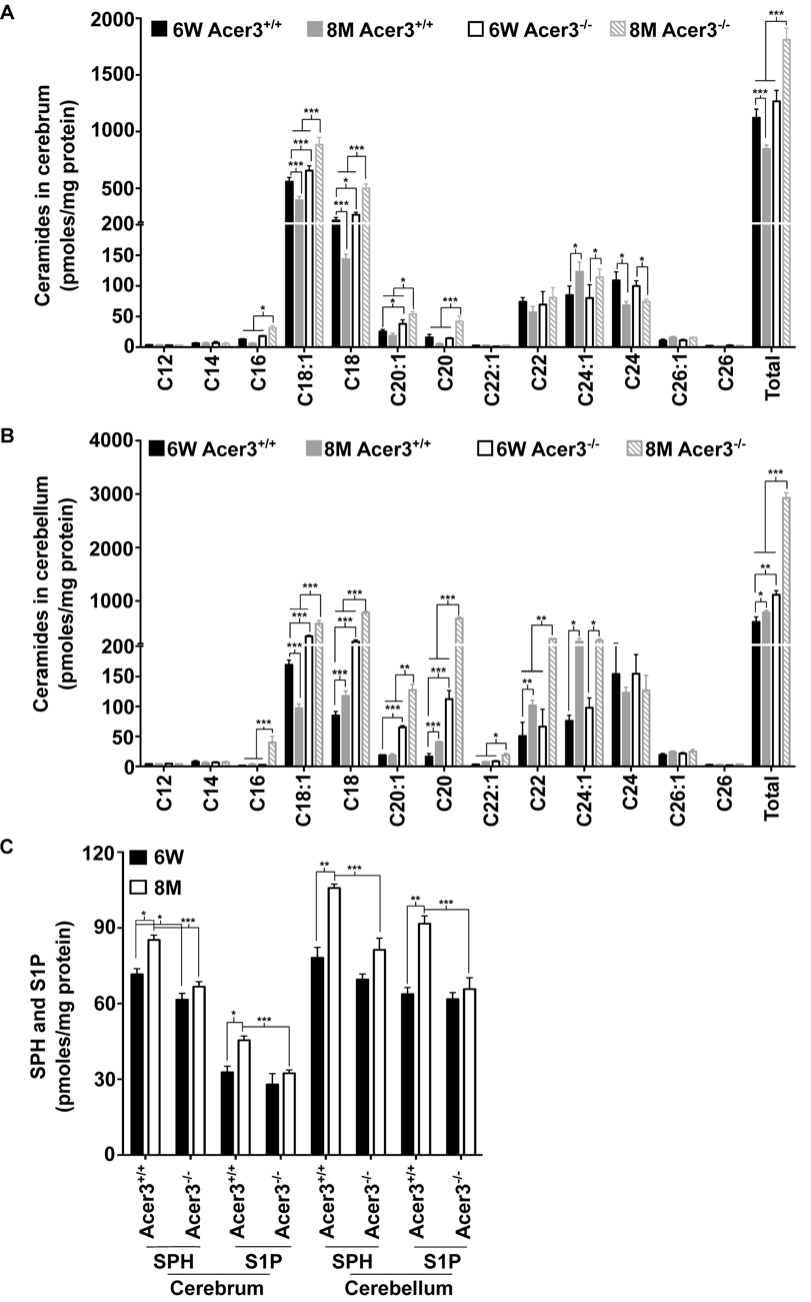
Acer3 upregulation is important for the homeostasis of ceramides, SPH, and S1P in aging brain. **A**-**B**. Levels of individual ceramide species and total levels of ceramides in the cerebrum (A) and cerebellum (B) of Acer3^-/-^ or Acer3^+/+^ mice at 8M or 6W of age. Note that C_18:1_-ceramide was significantly decreased in both the cerebrum and cerebellum of 8M-old Acer3^+/+^ mice compared to 6W-old Acer3^+/+^ mice and that this decrease was reverted in Acer3^-/-^ mice. **C**. SPH and S1P levels in the cerebrum and cerebellum in Acer3^+/+^ or Acer3^-/-^ mice at 6W or 8M of age. Note that both SPH and S1P levels were increased in both the cerebrum and cerebellum in Acer3^+/+^ mice at 8M of age compared to at 6M-old of age and that these age-dependent increases in SPH and S1P were reverted in Acer3^-/-^ mice. Total levels of sphingolipids were the sum of individual species of the same class. Data in A-C represent mean values ± SD, n = 3.

To investigate if Acer3 plays a role in regulating the homeostasis of ceramides and their sphingolipid derivatives in the brain during aging, we first measured the levels of ceramides in cerebral and cerebellar tissues from Acer3^+/+^ and Acer3^-/-^ mice at 6 weeks and 8 months of age using LC-MS/MS. In mice at 6 weeks of age, Acer3 knockout increased not only the levels of ULCCs (C_18:1_ and C_20:1_-ceramides) but also the levels of saturated LCCs (SLCCs; C_16:0_, C_18:0_, and/or C_20:0_-ceramides) in the cerebrum (Fig 5A), and to a greater extent in the cerebellum (Fig 5B). Acer3 deficiency increased the total levels of ceramides in the cerebellum (Fig 5B) and to a lesser extent in the cerebrum (Fig 5A) in 6-week-old mice. In mice at 8 months of age, Acer3 knockout further accumulated ULCCs and SLCCs in both the cerebrum and the cerebellum, while there was an accumulation of VLCCs (C_22_ and C_22:1_-ceramide) only in the cerebellum. The total levels of ceramides were therefore elevated in both brain regions, but were greater in the cerebellum (Fig 5A and 5B). It is worth noting that parallel to the upregulation of Acer3 in the brain, the levels of C_18:1_-ceramide were decreased in both the cerebrum and cerebellum in 8-month-old mice compared to 6-week-old wild-type mice. Importantly, this decrease was inhibited by Acer3 knockout, suggesting that Acer3 upregulation is important in prohibiting the aberrant accumulation of C_18:1_-ceramide in the aging brain.

Because ceramidases, including Acer3, catalyze the hydrolysis of ceramides into SPH, which in turn is phosphorylated to form S1P, Acer3 upregulation in the brain may increase the levels of both SPH and S1P in this tissue. To test this possibility, the levels of SPH and S1P were determined in the cerebrum or cerebellum in Acer3^+/+^ or Acer3^-/-^ mice at 6 weeks or 8-months of age using LC-MS/MS. The results showed that in mice at 6 weeks of age, Acer3 knockout significantly decreased the levels of SPH (by 14.10%) but not S1P in the cerebrum, whereas, in mice at 8 months of age, Acer3 knockout significantly decreased the levels of SPH (by 21.74% and 23.13% in the cerebrum and cerebellum, respectively) and S1P (by 28.77% and 28.27% in the cerebrum and cerebellum, respectively) (Fig 5C). It is worth noting that in line with the age-dependent upregulation of Acer3, the levels of both SPH and S1P were increased in either the cerebrum or cerebellum in 8-month-old Acer3^+/+^ mice compared to 6-week-old Acer3^+/+^ mice. These age-dependent effects were abolished by Acer3 knockout (Fig 5C), suggesting that age-related Acer3 upregulation is responsible for the increases in the levels of both SPH and S1P in the aging mouse brain.

**Fig 6:**
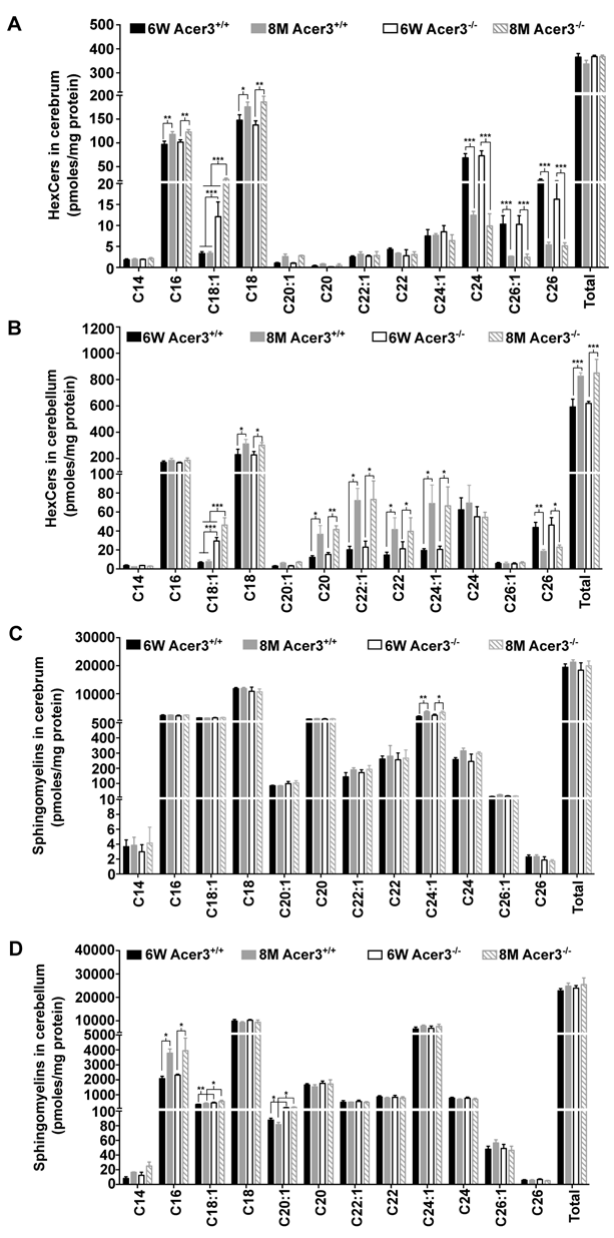
Acer3 upregulation is important for the homeostasis of complex sphingolipid in aging brain. **A** and **B.** Levels of individual monohexosylceramide (HexCer) species and levels of total HexCers in the cerebrum (A) and cerebellum (B) of Acer3^+/+^ or Acer3^-/-^ mice at 8M or 6W of age. Note that although causing a several-fold increase in the levels of C_18:1_-HexCer in both the cerebrum and cerebellum in mice at either age, Acer3 knockout did not alter the total levels of HexCers. **C** and **D**. Levels of individual sphingomyelins and total levels of ceramides in the cerebrum (C) and cerebellum (D) of Acer3^+/+^ or Acer3^-/-^ mice at 6W or 8Mof age. Note that Acer3 knockout only slightly increased the levels of C_18:1_ or C_20:1_-sphingomyelin in the cerebellum but not cerebrum in mice at either age without affecting the total levels of sphingomyelins in either brain region at either age. Total levels of sphingolipids were the sum of individual species of the same class. Data in A-D represent mean values ± SD, n = 3.

Ceramides can be incorporated into various complex sphingolipids. Since we observed an increase in the levels of brain ceramides in Acer3^-/-^ mice, we postulated that Acer3 deficiency may increase the levels of complex sphingolipids in the brain. To investigate this possibility, the levels of monohexosylceramides (HexCers) and sphingomyelins were determined in the cerebrum and cerebellum in Acer3^+/+^ and Acer3^-/-^ mice using LC-MS/MS. Acer3 knockout increased the levels of C_18:1_-HexCer, a very minor HexCer species, in the cerebrum (Fig 6A) and cerebellum (Fig 6B) in 6-week-old mice and to a greater extent in 8-month-old mice without affecting the levels of other HexCer species or total levels of HexCers (Fig 6A and 6B). These results suggest that Acer3 regulates brain C_18:1_-HexCer specifically and that its deficiency does not affect the total levels of this type of sphingolipids. As for sphingomyelins, the results showed that Acer3 knockout slightly but significantly increased the levels of C_18:1_ and C_20:1_-sphingomyelin in the cerebellum in mice at either 6 weeks or 8 months of age without affecting the total levels of sphingomyelins in this tissue and this effect was not observed in the cerebrum (Fig 6C and 6D). These results suggest that Acer3 regulates unsaturated long-chain sphingomyelins specially and that its deficiency does not affect the total levels of sphingomyelins.

### Acer3 knockout impairs motor coordination and balance

Ceramides have been shown to mediate various stress responses including apoptosis [43]. Since Acer3 deficiency caused an aberrant accumulation of LCCs in the brain, especially in the cerebellum, we hypothesized that Acer3 deficiency may lead to neuronal dysfunction. To assess the neurological consequences of Acer3 deficiency, Acer3^-/-^ and their littermate Acer3^+/+^ mice at different ages were subjected to a battery of behavioral tests.

General locomotor activity and movement pathways were evaluated by open field tests. Total walking distance, average velocity, corner area latency, and rearing activity were quantified. Movement pathways of 6-week-old, 8-month-old, and 12-month-old mice are illustrated in S2A Fig. After being placed in the center of an open field, both Acer3^+/+^ and Acer3^-/-^ mice ran to the edge, then explored the whole area starting from the edge to the central area. During the 5 min period, no significant difference was observed in total walking distance (S2B Fig), average velocity (S2C Fig), or corner area latency (S2D Fig) between Acer3^+/+^ and Acer3^-/-^ mice at 6 weeks, 8 months, or 12 months of age. However, rearing activity was significantly decreased in Acer3^-/-^ mice at 8 months and 12 months of age compared to their wild-type littermates, although such a difference was not observed in mice at 6 weeks of age (S2E Fig). These results suggest that Acer3 deficiency decreases open field rearing activity without affecting general locomotor activity in aging mice.

Motor coordination and synchrony were assessed by analyzing the footprint patterns as mice walked along a narrow corridor. The footprint patterns of Acer3^+/+^ and Acer3^-/-^ mice at 6 weeks, 8 months, and 12 months of age are illustrated in S3A Fig. At 6 weeks or 8 months of age, both Acer3^+/+^ and Acer3^-/-^ mice walked in a straight line with an even alternating gait and placed the hindpaw closed to the position where the ipsilateral forepaw had been in the previous step (S3B Fig). However, at 12 months of age, although both Acer3^+/+^ and Acer3^-/-^ mice walked in a straight line with an even alternating gait, their footprints of the hindpaw and ipsilateral forepaw did not overlap as perfectly as the other age groups (S3A Fig). The resulting footprint patterns were analyzed quantitatively by measuring stride length, hindpaw step width, forepaw step width, and the extent of overlap between the forepaws and hindpaw placement measured by the distance between the footprints. As shown in S3A Fig, Acer3^+/+^ and Acer3^-/-^ mice exhibited similar stride lengths and step widths at each age. There was no significant difference in the front/hind footprint overlapping between Acer3^-/-^ mice and their WT littermates at each age (S3B Fig), indicating that Acer3 knockout does not disrupt the uniformity of step alternation in mice at these ages.

To assess motor coordination and muscle strength, we first performed inverted wire hanging tests. The tests demonstrated that Acer3^-/-^ mice fell off the mesh upon inverting the wire mesh and did not attempt to walk on the inverted grid after they reached certain age (starting at 6 months of age). In contrast, their wild-type littermates continued grasping the mesh and also attempted to change foot placements on the inverted mesh (S1 Video). These results suggest that Acer3 deficiency may reduce grip strength and/or impair motor coordination.

Deficits in muscle strength were quantified by grip strength measurements. We found no significant difference in forepaw or hindpaw grip strength measurements between Acer3^+/+^ mice and Acer3^-/-^ mice at 4, 6, 8, or 12 months of age (S4A and S4B Fig), suggesting that Acer3 knockout does not compromise the muscle strength of mice up to 12 months of age.

**Fig 7:**
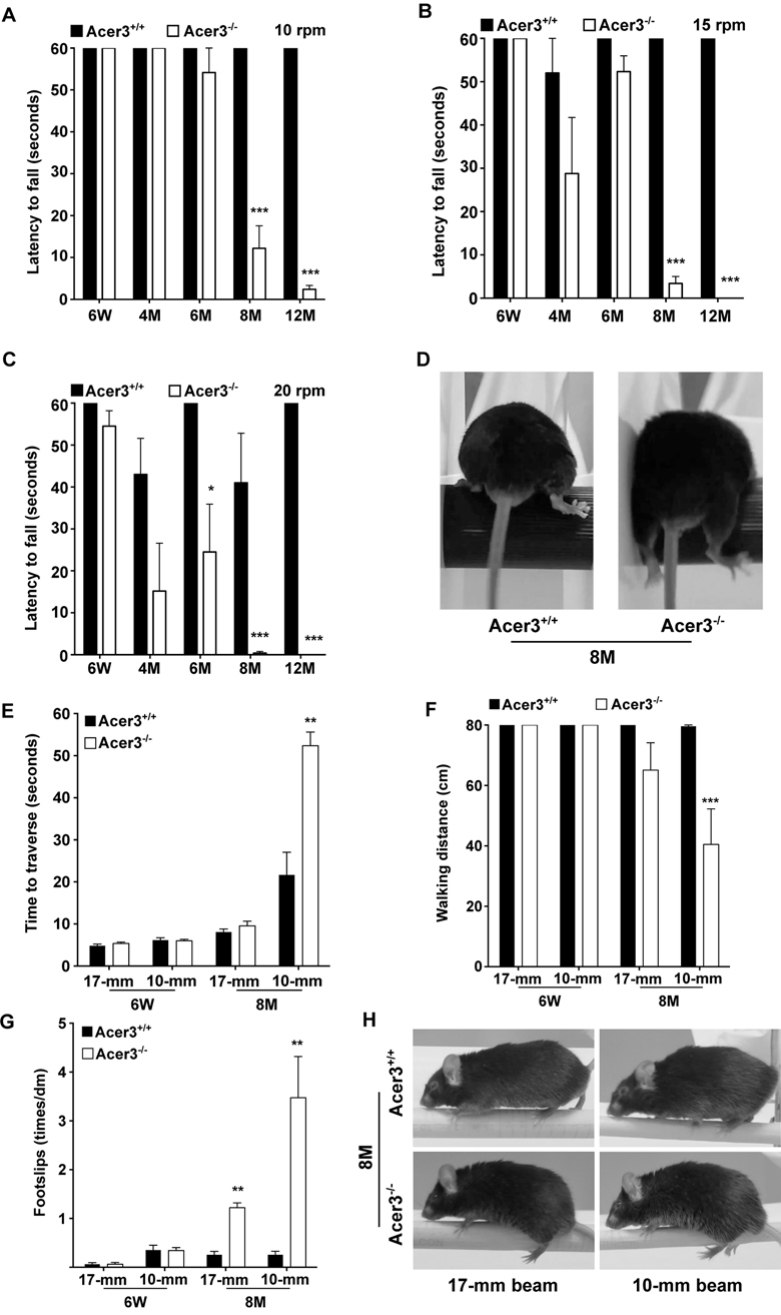
Acer3 knockout impairs motor coordination and balance capabilities in mice. **A**-**D**. Rotarod tests for motor coordination. Acer3^+/+^ and Acer3^-/-^ mice at 6W, 4M, 6M, 8M, or 12M of age were subjected to rotarod tests under 3 task difficulties—10, 15, and 20 rpm, respectively. Hindlimb step patterns in a representative Acer3^+/+^ and Acer3^-/-^ mouse at 8M of age at 20 rpm are displayed in D. Note that the hindpaws of Acer3^-/-^ mice, but not those of Acer3^+/+^ mice slipped off the rod. **E**-**H**. Beam walking tests for motor coordination and balance capabilities. Acer3^+/+^ and Acer3^-/-^ mice at 6W or 8M of age were subjected to beam walking tests under two task difficulties. The average of three trials were quantitatively analyzed for time to traverse the beam (E), walking distance (F), and foot-slips of hindpaws (G). Patterns of hindpaw contacting the beam during walking in a representative 8-month-old Acer3^+/+^ and Acer3^-/-^ mouse are displayed in H. Note the foot-slips for both beam walking conditions in the Acer3^-/-^ mouse. The data in A, B, C, E, F, and G represent mean values ± SD, n = 5–8. n.s., not significant.

We then performed rotarod tests to assess general motor coordination and balance. These tests were carried out with three task difficulties by varying the speed, and the latency to fall off the rod was recorded. We found that Acer3^-/-^ mice did not exhibit any significant difference in the latency to fall prior to 6 months of age when compared to its age-matched WT littermates (Fig 7A–7C). However, starting from 8 months of age, Acer3^-/-^ mice had difficulty staying on the rotarod at all testing speeds—walking on the rod was characterized by frequent bilateral foot-slips, decline in bilateral alternating movement patterns between the forelimb and hindlimbs and lack of balance, all of which led the Acer3^-/-^ mice to fall off the rod within a very short period. This was in striking contrast to the age-matched WT littermates who demonstrated significantly longer durations of walking times on the rotarod at all speed settings (Fig 7A–7D, S2 Video).

Next, beam walking tests were performed to compare the fine skill movement of the hindlimbs (paw placement on the beam) and balance capabilities of Acer3^+/+^ and Acer3^-/-^ mice. Two task difficulties were achieved by varying the diameter of cross-section of the beams. Traversing latency, walking distance, and foot-slip frequency were recorded during the beam test. Since rotarod tests revealed an obvious deficit in motor coordination and balance at 8 months of age in Acer3^-/-^ mice but not in younger mice, we chose 6-week-old and 8-month-old mice for these tests. At 6 weeks of age, both Acer3^+/+^ and Acer3^-/-^ mice traversed both types of beams with no difficulty and showed no difference in traversing latency (Fig 7E), walking distance (Fig 7F), or foot-slips (Fig 7G and 7H). At 8 months of age, Acer3 knockout mice made more foot-slips than their WT littermates on the 17-mm beam although they could still traverse the beam (Fig 7H). However, at 8 months of age, Acer3^-/-^ mice failed to complete all walking trials on the 10-mm beam, showed a significant increase in time to traverse the beam (Fig 7E), a marked decrease in average walking distance (Fig 7F), and exhibited more foot-slips (Fig 7G and 7H) compared to their WT littermates (S3 Video).

**Fig 8:**
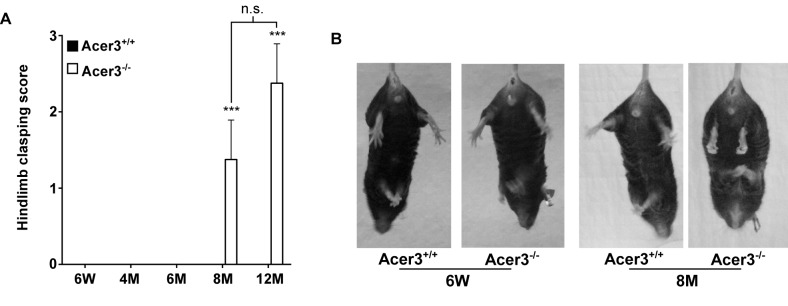
Acer3 knockout induces hindlimb clasping phenotype in mice. **A** and **B**. Hindlimb clasping reflex tests. Hindlimb clasping reflex was scored in Acer3^+/+^ and Acer3^-/-^ mice at 6W, 4M, 6M, 8M, and 12M of age (A). At 8M of age, Acer3^+/+^ splayed their hindlimbs outward and away from the abdomen similar to what was seen in the 6W mice, whereas Acer3^-/-^ mice retracted their hindlimbs toward the abdomen into a clasping reflex (B). The data in A represent mean values ± SD, n = 5–8. n.s., not significant.

Finally, the hindlimb clasping reflex was tested to determine if Acer3^-/-^ mice exhibit any neuropathology. Acer3^+/+^ and Acer3^-/-^ mice were suspended by the base of the tail for 10 s, and the duration of hindlimb clasping was scored. At 6 weeks to 6 months of age, both Acer3^+/+^ and Acer3^-/-^ knockout mice splayed their hindlimbs outward and away from the abdomen with no clasping reflex (Fig 8A). Starting at 8 months of age, while Acer3^+/+^ mice exhibited the same hindlimb action with no clasping reflex as the 6-week-old mice, Acer3^-/-^ mice retracted their hindlimbs toward the abdomen with a significantly higher hindlimb clasping reflex score than Acer3^+/+^ mice (Fig 8A and 8B).

Collectively, the behavioral tests suggest that Acer3 knockout in mice at 8 months of age drastically impairs overall motor coordination, skilled hindlimb function and balance capabilities without affecting general locomotor activity.

### Acer3 knockout induces premature degeneration of PCs

**Fig 9:**
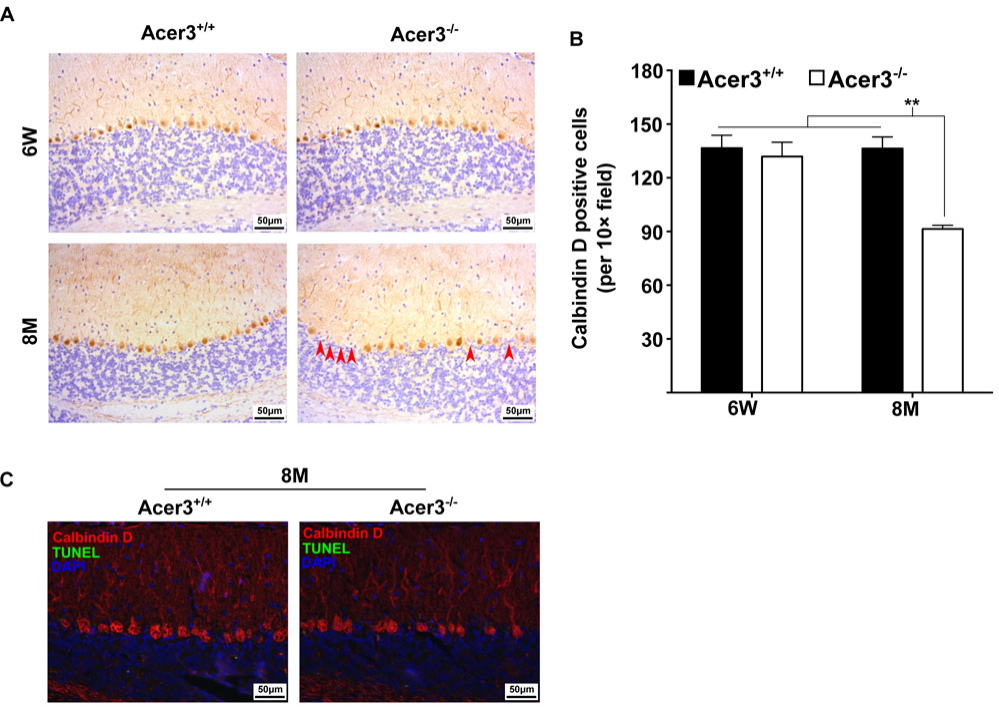
Acer3 knockout induces premature degeneration of PCs. **A** and **B**. PC loss in Acer3 knockout mice at 8M of age. Immunostaining of cerebellar sagittal sections with antibody against calbindin D-28K, a PC marker (A). Red arrowheads indicate the regions where PCs were lost. Quantification of PCs (B). Images in A are the results from a representative mouse in each group. **C**. TUNEL assays for apoptosis in the cerebellum from Acer3^+/+^ and Acer3^-/-^ mice. The cerebellar sections of Acer3^+/+^ and Acer3^-/-^ mice at 8M of age were co-stained with the TUNEL assay reagent (green fluorescence) and anti-calbindin D28K antibody (red fluorescence). The images in A and C are the results from a representative mouse in each group. The data in B represent mean values ± SD, n = 4.

The impairment of motor coordination and balance capacity reflects a dysfunction of the cerebellum [44]. PCs are the exclusive output neurons in the cerebellar cortex [45] and are shown to be sensitive to age-related damage in the cerebellum [45,46]. These observations prompted us to test if Acer3 deficiency caused pathological effects on PCs in this brain region. We examined PCs in Acer3^+/+^ and Acer3^-/-^ mice at 6 weeks or 8 months of age by immunohistochemical (IHC) staining with an antibody against calbindin D-28K, a PC marker. As shown in Fig 9A, calbindin D staining revealed well-arranged PCs between the molecular layer and granular layer of the cerebellum in both Acer3^+/+^ and Acer3^-/-^ mice at 6 weeks of age with a similar cell number. At 8 months of age, while Acer3^+/+^ mice still maintained a similar number and pattern of PCs to 6-week-old mice, Acer3^-/-^ mice exhibited a significant decrease in the number of PCs compared to their WT littermates and young animals (Fig 9A and 9B). Since Acer3 deficiency results in the accumulation of brain ceramides, which have been suggested to be pro-death bioactive lipids [47], we investigated if Acer3 deficiency induces apoptosis in PCs by performing terminal deoxynucleotidyl transferase dUTP nick-end labeling (TUNEL) assays. Interestingly, no apoptotic PCs were seen in the cerebellum of either 8-month-old Acer3^-/-^ mice or their WT littermates (Fig 9C). These results suggest that Acer3 knockout induces premature degeneration of PCs through apoptosis that escapes from detection by TUNEL assays or through a mechanism other than apoptosis.

### Acer3 knockout does not affect myelination

**Fig 10:**
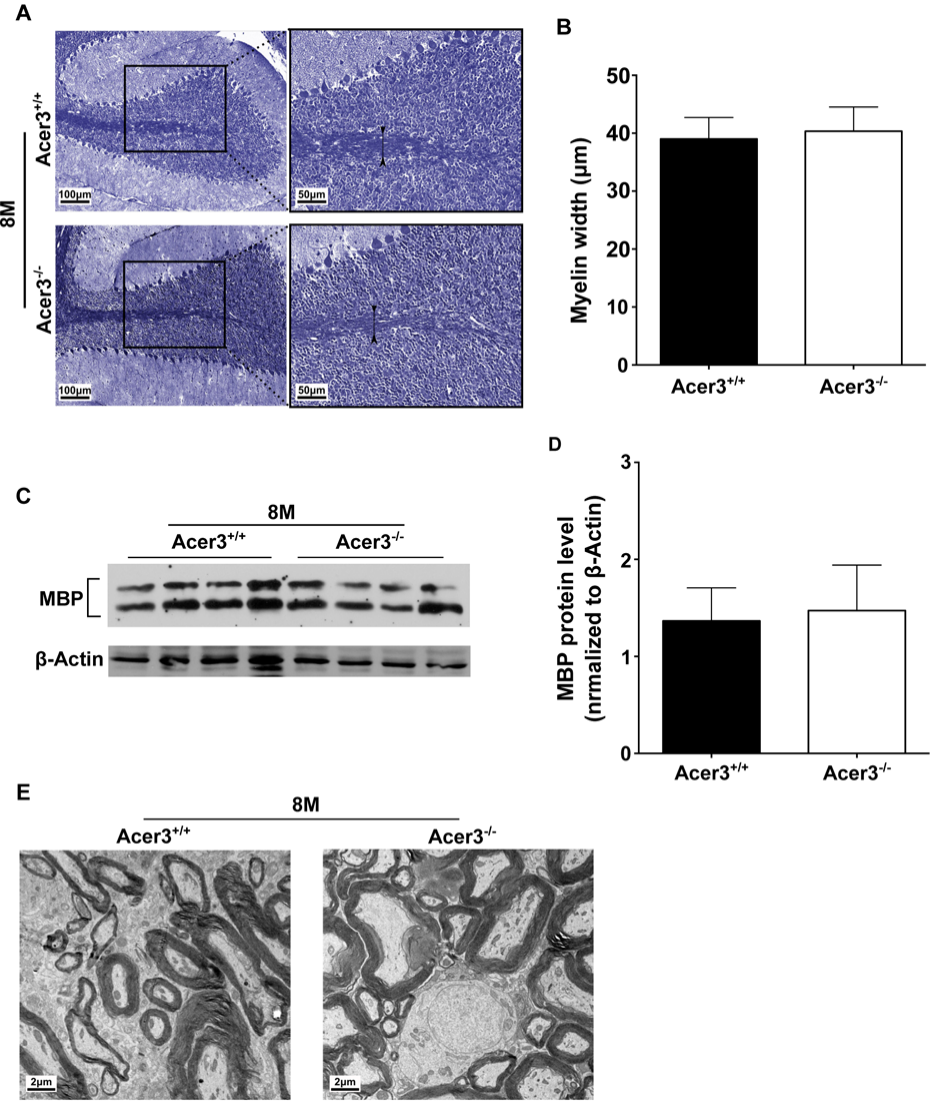
Acer3 knockout does not affect myelination. **A**-**D**. Cerebellar sections of Acer3^+/+^ and Acer3^-/-^ mice at 8M of age were stained with Luxol fast blue (A). The myelin width indicated by black arrowhead was measured in the images of the cerebellar sections stained with Luxol fast blue (B). MBP protein levels in cerebellar homogenates were analyzed by immunoblotting (C) and quantified (D). **E**. Electron microscopy of the ultrastructure of myelin sheaths in the cerebellum of Acer3^+/+^ and Acer3^-/-^ mice at 8M of age. The image in A or E represents the results from one of four mice in each group. The data in B and D represent mean values ± SD, n = 4.

Because dyshomeostasis of sphingolipids could lead to a defect in myelination, which has been linked to various neurological disorders including cerebellar ataxia [48,49], we tested if Acer3 deficiency impaired myelination in the cerebellum in mice at 8 months of age. Luxol fast blue staining showed that the width of myelinated tracts was not altered in Acer3^-/-^ mice as compared to Acer3^+/+^ mice (Fig 10A and 10B). Consistently, Western blot analyses found no difference in the levels of myelin basic protein (MBP) in the cerebellum between Acer3^+/+^ and Acer3^-/-^ mice (Fig 10C and 10D). Electron microscopy revealed that Acer3 deficiency did not alter the ultrastructure of myelin sheaths in the cerebellum (Fig 10E). These results suggest that Acer3 deficiency does not affect myelination.

## Discussion

Brain ceramide levels are increased in aging-associated conditions, such as Alzheimer’s disease (AD) [5,50] and during normal rodent aging [5]; thereby exerting pathological effects on brain functions [41,51]. On the other hand, S1P levels are decreased in the brain with AD [6,52]. Recent animal studies have demonstrated that a reduction in the levels of brain S1P is partly responsible for degeneration of PCs in a mouse model of Niemann–Pick disease type C (NPC) [4]. However, much remains unclear about how the homeostasis of ceramides and S1P in the brain is sustained during the normal aging process. In this study, we demonstrate for the first time that age-dependent upregulation of Acer3 plays a key role in sustaining the homeostasis of both ceramides and S1P in the aging mouse brain; thus protecting PCs from premature degeneration and hence from cerebellar ataxia.

Several studies have demonstrated that the levels of ceramides are elevated in tissues of aged rodents [53–55], including the brain [5], but it remains unclear whether this is also true in the aging brain of middle-aged mice. Middle age has been largely understudied in the context of aging, but investigating this life stage may be especially important for our understanding of aging. To this end, we measured the levels of ceramides in the brain in middle-aged mice versus young adult mice through a lipidomic approach. We demonstrate that the total levels of ceramides are moderately lower in the cerebrum but slightly higher in the cerebellum in middle-aged mice compared to young mice (Fig 5A and 5B). Interestingly, we observed that the levels of SPH and S1P were much higher in both brain regions in middle-aged mice than in young mice (Fig 5C). These results suggest that an aberrant accumulation of ceramides does not occur in the mouse brain at least up to the middle age (8 months) although there are age-associated increases in the levels of both SPH and S1P.

Another novel finding of our study is that Acer3 upregulation prevents abnormal accumulation of ceramides and increases the levels of their metabolites SPH and S1P in the aging brain. It has been reported previously that alkaline ceramidase activity is increased with age in the mouse brain [42]; here we identify the alkaline ceramidase isozyme that encodes the increased activity. We demonstrate that the mRNA levels of Acer3, but not other alkaline ceramidases, are upregulated with age in the mouse brain (Fig 1A). We confirmed that alkaline ceramidase activity on NBD-C_12_-PHC, a preferred substrate for Acer3 but a poor substrate for the other ceramidases, was also increased with age in both the cerebrum and cerebellum (Fig 1C). The underlying mechanism for such age-dependent Acer3 upregulation however needs further investigation.

Consistent with Acer3 upregulation, there is a decrease in levels of the brain C_18:1_-ceramide, a preferred substrate of Acer3, along with concurrent increases in SPH and S1P levels in middle-aged mice compared to young adult mice (Fig 5A and 5B); indicating that Acer3 upregulation has a role in preventing C_18:1_-ceramide accumulation in the aging brain. This is supported by our finding that Acer3 deficiency increased the levels of C_18:1_-ceramide in the brain. C_18:1_-ceramide is abundant in the brain whereas it is scarce in the non-central nervous system (CNS) tissue such as liver and lungs under basal conditions (S1 Table). Ceramides with different acyl-chains are synthesized by the action of different CerSs [56]. Ginkel *et al*. have demonstrated that knocking out CerS1 markedly lowered the levels of C_18:1_-ceramide in the mouse brain, thereby implying that CerS1 is a CerS isozyme responsible for the biosynthesis of this particular ceramide species in addition to SLCCs [48]. CerS1 is mainly and highly expressed in the brain [16], explaining why the brain has the highest content of C_18:1_-ceramide among other tissues (Fig 5A and 5B, and S1 Table). This tissue-specific ceramide composition explains why Acer3 knockout dramatically increased the levels of C_18:1_-ceramide and total levels of ceramides in the brain (Fig 5A and 5B), but did so only modestly in other tissues (S1 Fig and S1 Table). Unexpectedly, Acer3 knockout also caused a marked increase in the levels of other long-chain ceramides in the brain (C_16_, C_18:0_, C_20:0,_ C_22:0,_ and C_22:1_-ceramide), although these are not the most preferred substrates of Acer3 (Fig 5A and 5B). We propose that some of ULCCs accumulated due to Acer3 deficiency were converted by other ceramidases to SPH, which was in turn re-acylated to form other ceramides by the action of different CerS isozymes.

Our study demonstrates that Acer3 deficiency also led to an increase in the levels of C_18:1_- and/or C_20:1_-HexCers and sphingomyelins in the brain, most likely due to an accumulation of common precursors such as the ULCCs. Acer3 deficiency also increased the levels of these complex sphingolipid species in non-CNS tissues (S1 Table). However, Acer3 deficiency did not increase the total levels of these complex sphingolipids in all the tissues examined, probably because these ULC complex sphingolipids are minor species in these tissues (S1 Table) [36]. Similarly, total levels of more complex glycosphingolipids, such as gangliosides and sulfatides, which are derived from HexCers, are not expected to be significantly affected by Acer3 deficiency although C_18:1_ or C_20:1_-gangliosides or sulfatides may be increased.

To our knowledge, our study is the first to discover an age-dependent increase in the levels of both SPH and S1P in the mouse brain (Fig 5C). Acer3 knockout nearly abolished the age-dependent increase in the levels of both brain SPH and S1P (Fig 5C), suggesting that Acer3 upregulation is solely responsible for the increased generation of these bioactive lipids in the aging brain although other ceramidase activities were also found to be increased with age in the brain [42]. Acer3 deficiency also led to a slight decrease in the levels of both SPH and S1P in the lungs but not in the liver (S1 Table), suggesting that Acer3 has a minimum role in regulating SPH and S1P levels in the non-CNS tissue likely due to the scarcity of its substrates, ULCCs, in these tissues.

Acer3 deficiency mainly affects brain function without inducing any major pathological effect on other tissues that we examined (S5 and S6 Figs). In the brain, Acer3 deficiency also causes a region-specific effect on the homeostasis of sphingolipids, altering the levels of ceramides, SPH, and S1P to a much greater extent in the cerebellum than in the cerebrum. This is consistent with the fact that Acer3 expression is much higher in the cerebellum than in the cerebrum (Fig 1B and 1C). We showed that Acer3 knockout impaired motor coordination in mice (Fig 7) without affecting general locomotor activity (S2 Fig) or muscle strength (S4 Fig). Mechanistic studies suggest that Acer3 deficiency impairs motor coordination at least in part due to the degeneration of PCs (Fig 9) although dysfunction of other types of neurons in different brain regions may also contribute to this phenotype.

Interestingly, the degeneration of PC and impairment in motor coordination were observed in middle-aged knockout, but not younger mice. These data coincide with the finding that Acer3 deficiency altered the homeostasis of ceramides and their metabolites in the cerebellum to a greater extent in middle-aged mice than in young mice. It is worth noting that Acer3^+/-^ mice at 8 months of age behaved similarly to Acer3^+/+^ mice at the same age in the rotarod (S7A Fig) or hindlimb clasping test (S7B Fig), suggesting that a single copy of the *Acer3* gene is sufficient to maintain motor coordination function. This is consistent with the finding that there is no difference in the content of any aforementioned brain ceramides between Acer3^+/+^ and Acer3^+/-^ mice (S7D and S7E Fig), suggesting that Acer3 is a haplosufficient gene in maintaining the homeostasis of sphingolipids in the brain.

The close correlation between the dyshomeostasis of sphingolipids and pathology strongly suggests that Acer3 deficiency causes PC degeneration by altering the levels of sphingolipids in PCs and/or other brain cell types that interact with PCs. Ceramides, especially SLCCs, have been shown to be pro-death bioactive lipids [57], whereas S1P functions as a pro-survival bioactive lipid [58]. The PC degeneration by Acer3 deficiency is probably due to both an aberrant rise in ceramides and a significant decline in S1P in the cerebellum.

Because there was no difference in the number of PCs between Acer3^+/+^ and Acer3^-/-^ mice at a young age, it appears that Acer3 deficiency does not affect the development of PCs. We surmise therefore that the PC loss in middle-aged mice is likely due to cell death. Since we did not observe apoptosis in all PCs in middle-aged Acer3^-/-^ mice (Fig 9C), the PC loss may be through a non-apoptotic cell death mechanism or that the neuronal death pathway operates gradually in the Acer3^-/-^ mice, thus eluding detection. Interestingly, recent studies have also demonstrated that inhibiting biosynthesis of LCCs in the mouse brain due to mutation [59] or deficiency [48] in CerS1 leads to progressive cerebellar ataxia due to the degeneration of PCs; thereby suggesting that LCCs are essential for the survival of PCs, although their aberrant increase is also detrimental to these cells.

Whether the PC degeneration resulting from Acer3 deficiency is a cell-autonomous or nonautonomous trait remains unclear because the global gene knockout approach cannot define a cell-type-specific effect of Acer3 deficiency on the dyshomeostasis of sphingolipids. Because ULCCs are mainly synthesized by CerS1, which is expressed in neurons rather than glia cells in the brain [48], we postulate that PC degeneration due to Acer3 deficiency may be a direct pathological effect of the dyshomeostasis of sphingolipids in neurons rather than in glial cells. However, to resolve this uncertainty requires a genetic approach by which Acer3 is ablated specifically in PCs. This approach is currently being explored in our laboratory.

Although SPH, similar to ceramides, has been implicated in apoptosis in various cell types [47], recent findings demonstrated that SPH can also regulate synaptic vesicle fusion and exocytosis [60]. Whether Acer3 deficiency adversely affects the neuronal synapse function by inhibiting SPH generation requires further investigations. An aberrant accumulation of glycosphingolipids or sphingomyelins has been shown to impair neuronal function in patients and in the mice with deficiency or mutations in enzymes responsible for their breakdown [61,62]. As discussed earlier, Acer3 deficiency only slightly increased the levels of some minor species of monohexosylceramides and sphingomyelins without affecting their total levels, so the pathological effects observed in Acer3 null mice may not be due to the minor alterations in these complex sphingolipids. Recently, alterations in gangliosides and/or sulfatides have been suggested to be detrimental to cerebral and cerebellar myelination, which is key to neuronal function [48]. Due to an insignificant effect on these complex sphingolipids, Acer3 deficiency is unlikely to impair myelination and myelination-associated neuronal function. Consistent with this view, we showed that Acer3 deficiency does not affect myelination and general locomotion activity. However, there is a possibility that an increase in C_18:1_ or C_20:1_-sphingomyelin and complex glycosphingolipids may adversely affect other neuronal functions, thereby contributing to the impairment in motor coordination and balance.

This study and previous studies [37,41] are beginning to establish the proposition that different ceramidases have distinct roles in regulating the homeostasis of ceramides *in vivo*. In addition to Acer3 knockout mice, mouse models deficient in Asah1 [41] and Asah2 [37], respectively, have been established. Different from Acer3 deficiency, Asah2 deficiency mainly alters the homeostasis of ceramides and SPH in intestines but not in the mouse brain [37]. Consistently, the deficiency of this ceramidase has not yet been reported to have any neurological phenotype in mice. We do not yet know the role for Acer1 or Acer2 in regulating the homeostasis of ceramides in the CNS, but a mouse model deficient in Acer1 or Acer2 is being developed in our laboratory. Compared to Acer3 knockout mice, Asah1 knockout mice have shown more profound phenotypic changes. Li *et al* have demonstrated that global knockout of Asah1 leads to embryonic lethality [63]. The same group has shown that knocking out Asah1 specifically in ovary caused apoptosis of oocytes, resulting in female infertility [64]. Recently, Alayoubi *et al* revealed that mice carrying a mutated Asah1 gene, which encodes an acid ceramidase mutant with minimal enzymatic activity, had necrosis in various tissues and died at a young age [41]. Asah1 deficiency markedly increased the total levels of ceramides in various tissues whereas Acer3 deficiency did so mainly in the brain. The distinct pathological consequences resulting from Acer3 and Asah1 deficiency are likely due to their distinct roles in regulating the hydrolysis of different ceramide species in tissues and in different subcellular compartments.

In conclusion, the present study demonstrates that the age-associated upregulation of Acer3 plays a crucial role in the survival and/or function of PC in aging mice via maintaining the homeostasis of ceramides and their metabolites in the cerebellum. The Acer3 knockout mouse model can serve as an invaluable tool for elucidating the pathological role for dysregulation of ceramides in neurodegenerative diseases in humans.

## Materials and Methods

### Mice

All mice were housed under a constant room temperature (22°C), humidity level (55%), and a 12-h light:12-h dark cycle with food (W.F. Fisher & Son; Somerville, NJ) and water available *ad libitum*. The procedures for the generation of Acer3 null mice were approved under the number AR2123 by the Institutional Animal Care and Use Committee (IACUC) of the Medical University of South Carolina, SC, USA, and other animal studies were approved under the number 580863 by the IACUC of Stony Brook University, Stony Brook, NY, USA. All the animal studies reported in this study were conducted according to the Animal Research: Reporting In Vivo Experiments (ARRIVE) guidelines, developed by the National Centre for the Replacement, Refinement & Reduction of Animals in Research (NC3Rs).

### Generation of Acer3 null mice

The mouse Acer3 gene consists of 11 exons (Fig 2A). An Acer3 targeting vector was constructed so that upon homologous recombination between the targeting vector and the WT Acer3 allele, exon 8 of the Acer3 gene was replaced by the neomycin resistant gene (*Neo*) cassette (Fig 2A). To construct the *Acer3* targeting vector, with PCR, we amplified a 1.3 kb fragment (the short arm) and 5.8 kb fragment (the long arm) upstream and downstream, respectively, of exon 8 from the genomic DNA of 129 SvEv embryonic stem (ES) cells. The short and long arms were inserted upstream and downstream, respectively, of the *Neo* gene in the vector OSDUPDEL (Fig 2A), a gift from Dr. Oliver Smithies at the University of North Carolina at Chapel Hill, Chapel Hill, North Carolina. The resulting Acer3 targeting construct was linearized by the restriction enzyme *Not1* and transfected into 129SvEv ES cells by electroporation as described [65]. Transfected ES cells were cultured in medium containing G418 (200 μg/ml), and the resulting G418-resistant ES clones in which one copy of the Acer3 alleles was correctly targeted by *Neo* were screened by Southern blot analyses (Fig 2B), expanded, and injected into mouse C57BL/6J blastocysts using standard procedures as described [65]. The injected blastocysts were transplanted into the uteri of pseudopregnant C57BL/6J mice, and the resulting chimeric mice were crossed to WT C57BL/6J mice to generate Acer3^+/-^ mice. Acer3^+/-^ mice with a mixed genetic background were backcrossed to WT C57BL/6J mice for 16 generations to obtain Acer3^+/-^ mice with the sole C57BL/6J genetic background. These heterozygous mice were inbred to generate Acer3^-/-^ mice and their Acer3^+/+^ littermates, which were used for further studies. DNA was isolated from mouse tail clips as described [66] and subjected to PCR-based genotyping using two different primer pairs (F1/B1 and F1/B2) as shown in Fig 2A. Acer3^+/+^, Acer3^+/-^, and Acer3^-/-^ mice were identified according to the PCR product patterns as shown in Fig 2C.

### Protein concentration determination

Protein concentrations were determined with bovine serum albumin (BSA) as a standard using bicinchoninic acid (BCA) protein determination kits (Thermo Scientific; Waltham, MA) according to the manufacturer’s instructions.

### Alkaline ceramidase activity assays

Tissues were collected from mice euthanized by CO_2_ suffocation followed by cervical dislocation, rinsed with phosphate buffered saline (PBS), snap-frozen in liquid nitrogen, and stored at -80°C. The tissues were homogenized on ice with an electric tissue tearor (Biospec Products; Bartlesville, OK) in Buffer A (25 mM Tris-HCl, pH 7.4, 150 mM NaCl and 0.25 M sucrose) supplemented with a protease inhibitor mixture (Roche; Indianapolis, IN). After brief sonication, the tissue homogenates were centrifuged at 1,000 *g* at 4°C for 5 min to sediment nuclei and tissue debris, and the resulting supernatants were centrifuged at 100,000 *g* at 4°C for 45 min to pellet all cell membranes, which were resuspended by brief sonication in Buffer B (25 mM Tris, pH7.4, 5mM CaCl_2_ and 150 mM NaCl). Membrane homogenates (20 μg or 40 μg of protein per tissue) were measured for alkaline ceramidase activity using NBD-C_12_-PHC as a substrate by a thin layer chromatography (TLC) method [30] or using regular ceramides as substrates by liquid chromatography tandem-mass spectrometric analysis (LC-MS/MS) method [35]. Briefly, NBD-C_12_-PHC or a regular ceramide was dispersed by water bath sonication in Buffer C (25 mM glycine-NaOH, pH 9.4, 5 mM CaCl_2_, 150 mM NaCl, and 0.3% Triton X-100). The lipid-detergent mixtures were boiled for 30 s and chilled on ice immediately to form homogeneous lipid-detergent micelles, which were mixed with an equal volume of membrane homogenates suspended in Buffer B. Enzymatic reaction was carried out at 37°C for 40 min, and stopped by heating at 100°C on a heating block. The reaction mixtures were completely dried, cooled down to room temperature, and dissolved in chloroform/methanol (2:1). For the TLC method, 20 μl of each reaction mixture was spotted onto TLC plates, which were developed in a solvent system consisting of chloroform, methanol, and 25% ammonium hydroxide (90:30:0.5). The TLC plates were dried and scanned by an imaging system (Typhoon FLA 7000, GE Healthcare Life Sciences; Pittsburgh, PA) set at the fluorescence mode. The fluorescent band of NBD-C_12_-fatty acid (NBD-C_12_-FA) released from NBD-C_12_-PHC was identified according to the standard NBD-C_12_-FA spotted on the same TLC plate. The content of NBD-C_12_-FA in each reaction was determined according to a standard curve generated from known concentrations of the standard NBD-C_12_-FA. For the LC-MS/MS method, lipids were extracted from dried reaction mixtures with chloroform and methanol. Sphingoid bases in the lipid extracts were determined by LC-MS/MS with D-e-C_17_-SPH as an internal standard as described [35].

### LC-MS/MS sphingolipid analysis

Tissues collected from Acer3^+/+^ or Acer3^-/-^ mice were homogenized on ice as previously described in buffer E (25 mM Tris–HCl, pH 7.4, 150 mM NaCl, 1mM EDTA, and 1mM EGTA). Lipids from tissue homogenates (2 mg protein per sample) were extracted with ethyl acetate/isopropanol/water (60/30/10, v/v). The lipid extracts were dried under N_2_ gas stream and reconstituted in methanol, and sphingolipids were determined by LC-MS/MS performed on a TSQ 7000 triple quadrupole mass spectrometer (Thermo Finnigan; Ringoes, NJ) as described [35]. Amounts of sphingolipids in different samples were normalized to protein contents.

### Immunohistochemistry (IHC)

Mice were deeply anesthetized with urethane (1.5 g/kg) and perfused transcardially with PBS followed by 4% paraformaldehyde (PFA) as described [59]. Brains, left lateral lobes of liver, and left lungs were removed and post-fixed in 4% PFA before dehydration and embedding in paraffin. Sagittal sections from one individual mouse brain were stained with an antibody against the PC marker calbindin-D-28K (Sigma-aldrich, St. Louis, MO) using a Histostain-Plus IHC staining kit (Invitrogen; Grand Island, NY). Cells stained positive for calbindin D in the cerebellum were enumerated from 3 random 10× fields of view under an Imager M2 microscope (Zeiss; Thornwood, NY) in a blind manner, and three serial sections were scored and the average score of each mouse cerebellum was calculated. Lung and liver tissue sections were stained with anti-Ki67 antibody (Biocare Medical; Concord, CA) using a Histostain-Plus IHC staining kit (Invitrogen; Grand Island, NY). Ki67-positive cells were scored under an Imager M2 microscope (Zeiss; Thornwood, NY) in a blind manner. In lungs, Ki67-positive cells in the bronchial epithelium and alveoli were scored from 4 random 10× and 4 random 20× microscopic fields of view, respectively, and the percentage of Ki67-positive cells was calculated. In liver, Ki67 positive cells were enumerated from 4 random 10× fields of view.

### TUNEL assays and immunofluorescent staining (IFS)

After mice were perfused with 4% PFA under deep anesthesia, brains were removed and cut into sagittal slices on a cryostat. Cryosections were subjected to TUNEL assays using an *in situ* cell death detection kit (Roche; Indianapolis, IN) according to the manufacturer’s instructions. After TUNEL staining, sections were subjected to IFS with calbindin D-28K antibody (Sigma-aldrich, St. Louis, MO). TUNEL and IFS were analyzed under an Imager M2 microscope (Zeiss; Thornwood, NY) in a blind manner. Lung and liver paraffin-embedded sections were prepared as described above and subjected to TUNEL assays using TACS^®^ 2 TdT diaminobenzidine kit (Trevigen; Gaithersburg, MD) according to the manufacturer’s instructions. TUNEL-positive cells were scored in a blind manner as described earlier. In the lung sections, positive cells in bronchial epithelium and in alveoli were counted from 4 random 10× and 4 random 20× microscopic fields of view, respectively. In the liver sections, positive cells were enumerated from 4 random 10× fields of view.

### RNA extraction and qPCR

RNAs were extracted from fresh brain tissues using RNeasy mini kits (Qiagen; Valencia, CA) according to the manufacturer’s instructions. The RNAs were reversely transcribed into cDNAs, which were subjected to qPCR analyses as described [67] using primer pairs specific to each of the following genes: *Acer1* (5’-ATGCTCATAGGTCTGTTCTC-3’ and 5'-AGTGGTTATAGTTACCAGGC-3’), *Acer2* (5’-GTGTGGCATATTCTCATCTG-3’ and 5’-TAAGGGACACCAATAAAAGC-3’), *Acer3* (5’-GTGTGGCATATTCTCATCTG-3’ and 5’-TAAGGGACACCAA TAAAAGC), and *β-Actin* (5’- GATGTATGAAGGCTTTGGTC-3’ and 5’-TGTGCACTTTTATTGGTCTC-3’). qPCR was performed on an ABI Prism 7000 sequence detection system and mRNA levels for each gene were analyzed with the ABI Prism 7000 software (Applied Biosystems). Relative mRNA levels of Acer1, Acer2, or Acer3 were estimated using ΔΔCt method as described [68] with β-Actin as internal control.

### Luxol fast blue staining

Paraffin cerebellar sections from 8-month-old mice were subjected to Luxol fast blue staining and myelin sheaths were assessed as described [59,69]. Briefly, images were taken using a black and white camera then pseudo-colored using Imagepro software. The width of myelinated tracts was measured as an indicator for alteration of myelin.

### Protein extraction and western blot analyses

Mouse tissues were minced with a razor blade and homogenized as previously described in Buffer F (50 mM Tris-HCl pH 7.5, 250 mM NaCl, 1mM EDTA, 1mM EGTA, 1% Triton X-100, and 1% SDS) supplemented with protease inhibitor mixture (Roche; Indianapolis, IN) and phosphatase inhibitor cocktail (Thermo Scientific; Waltham, MA). Proteins were separated on SDS-polyacrylamide gels and transferred onto nitrocellulose membranes, which were probed with the primary antibody against Ki67 (Biocare Medical; Concord, CA), proliferating cell nuclear antigen (PCNA), caspase 3 (Cell Signaling; Beverly, MA), cleaved caspase 3 (Cell Signaling; Beverly, MA), MBP (Sigma-aldrich; St. Louis, MO), or β-actin (Santa Cruz; Dallas, Texas). Band density was quantified by densitometry using NCBI ImageJ.

### Open field tests

Open field tests were performed as described [70] with slight modification. The open field consists of a square arena (40×40 cm), with a floor enclosed by continuously 25-cm-high walls. The animals were tested in this field during the light phase of their light/dark cycle. The test was initiated by placing a single mouse in the center of the arena and letting it move freely for 5 min. Mouse behavior was continuously videotaped by a video camera placed over the structure and then encoded using a continuous sampling method by Ethovision XT 7.0 (Noldus; Attleboro, MA). The arena was cleaned with ethanol after every test. A number of parameters were collected during the session. They comprised: (1) Velocity: the distance of the mouse moved per second (cm/s); (2) Distance: the distance (cm) the mouse moved in the five min of the test; (3) Latency to corner: the time (s) from when the mouse was first placed in the arena to when the mouse faced into a corner; (4) Rearing: the number of times the mouse reared on its hindpaws.

### Inverted wire hanging tests

Mice were placed in the center of a wooden-framed wire mesh, and the frame was inverted at a height of 35 cm above soft padding. Mice were observed for movement while hanging from the wire mesh, and the hang time was measured from the time the frame was inverted to the time the mouse fell. The maximum length of time the frame was inverted was one minute [71].

### Grip strength measurement

The muscular strength of mouse forepaws or hindpaws was measured using a grip strength meter (TSE Instruments International; Chesterfield, MO). Two attachments were used—a bar specific for mouse forepaws and a wire grid that allowed for simultaneous grip with all four paws. In both testing scenarios, the mouse was held by the base of its tail and allowed to firmly grab the attachment with either the forepaws, or with forepaws and hindpaws combined. A gentle and consistent tug backwards on the tail was applied to ensure it was firmly gripping the attachment before being quickly pulled off perpendicular to the force gauge in one motion [72,73]. The software for the meter displayed the peak force achieved during this motion. Five successive measurements were taken and the three highest measurements were averaged to give the strength score. Each mouse was given fifteen seconds of rest between each trial. The peak force (N) of each trial for the hindpaws was calculated by subtracting the peak force for the forepaws from the peak force of all four paws.

### Rotarod tests

Rotarod tests were performed using a rotarod device (Coulbourn Instruments; Whitehall, Pennsylvania). Mice were trained for 10 min, 6 days a week, for a total of 9 sessions. Each session included 3 trials. The first two trials (3 min each) consisted of assisted training where the mice were gently supported by the trainer’s fingers or hand while walking on the rod. The third trial was consisted of independent walking (for 4 min). For each trial, animals were trained at their maximum tolerable speed. Training was truncated at 25 rpm for both assisted and independent stepping. Mice that achieved independent stepping at 25 rpm before 9 sessions were still given the same training duration until 9 sessions were met. Note that this baseline training was crucial to ensure that mice were falling off the rod not because they were unfamiliar with the task, but because they were not able to meet the demands of the rotarod task. Mice were tested for two consecutive days immediately following their final training day. Each testing day consisted of three constant speeds; the latency to fall at each constant running speed was used as measures for rotarod tests [74,75] and the highest score of both days was recorded.

### Footprint tests

Footprint tests were performed as described [76]. Briefly, the hindfeet and forefeet of the mice were coated with blue and red nontoxic paints, respectively. Mice were then allowed to walk along a 40-cm-long, 10-cm-wide runway (with 15-cm-high walls) into an enclosed box. All mice had three training runs and were then given one testing run. A fresh sheet of white paper was placed on the floor of the runway for each run. The footprint patterns were analyzed for four parameters (all measured in centimeters): (1) stride length measured as the average distance of forward movement between each stride, (2) hindpaw step width measured as the average distance between left and right hind footprints, (3) forepaw step width measured as the average distance between and left and right front footprints, and (4) distance between ipsilateral forepaw and hindpaw footprints measured to indicate the extent of overlap between ipsilateral forepaws and hindpaws (paw overlap). For each parameter, three values were measured, excluding where the animal was initiating and finishing movement, respectively. The mean value of each set of three values was used in subsequent datum analyses.

### Beam walking tests

Beam walking tests were performed as described [76] with slight modification. Two customized round beams with diameters of 17 and 10 mm were used. The beams were placed horizontally, 50 cm above the bench surface, with one end mounted on a narrow support and the other end attached to an enclosed box (20×10×10 cm) into which mice could escape. During training, mice were placed at the start of the beam and trained over 3 days (4 trials per day) to traverse the beam to the enclosed box. Once the mice were trained, they received 3 consecutive trials on each beam, progressing from the 17-mm to 10-mm beam. Mice were allowed up to 60 s to traverse each beam. The time to traverse each beam was recorded. If a mouse fell off the beam, its latency was recorded as 60 s. The walking distance was measured for each trial. Mouse hindlimb movement was continuously monitored by a video camera, and the recorded videos were used for enumerating the times that the hindfeet of mice slip off each beam in each trial. For each parameter, the mean value of the three trials for each beam was used in subsequent datum analyses.

### Hindlimb-clasping tests

Hindlimb-clasping tests were carried out as described [77]. Mice were individually lifted by grasping their tails near the base and their hindlimb positions were observed for 10 s. If the hindlimbs were consistently splayed outward and away from the abdomen, it was scored as 0. If one hindlimb was retracted toward the abdomen for more than 5 s, it was scored as 1. If both hindlimbs were partially retracted toward the abdomen for more than 5 s, it was scored as 2. If its hindlimbs were entirely retracted and touching the abdomen for more than 5 s, it was scored as 3.

### Transmission electron microscopy (TEM)

Cerebella were dissected from mice perfused with 3.4% PFA/1.25% glutaraldehyde under deep anesthesia and sliced into 60 μm sagittal sections under a microtome [78]. The tissue sections were then processed for TEM as describe[79]. Briefly, the tissue sections were washed with 0.1 M sodium cacodylate buffer (pH 7.4) containing 2 mM CaCl_2_ and post-fixed for 1 h in a reduced osmium solution containing 2% osmium tetroxide, 1.5% potassium ferrocyanide 2 mM CaCl_2_ in 0.15 mM sodium cacodylate buffer (pH 7.4). After being washed with double-distilled water (ddH_2_O), the tissue sections were incubated with a 1% thiocarbohydrazide (TCH) solution in ddH_2_O for 20 minutes at room temperature (RT). After being washed with ddH_2_O, the tissue sections were fixed with 2% osmium tetroxide in ddH_2_O for 30 min at Rt, followed by 1% uranyl acetate (aqueous) overnight at 4°C. After being washed with ddH_2_O, the tissue sections were subjected to *en bloc* Walton’s lead aspartate staining in a 60°C oven for 30 min as described [80] After five 3-min rinses, the tissue sections were dehydrated sequentially in 20%, 50%, 70%, 90%, 100%, and 100% ice-cold ethanol (anhydrous), 5 min each, followed by anhydrous ice-cold acetone at RT for 10 min. The tissue sections were infiltrated with 30% Epon-Aradite for 2 h, 50% Epon-Aradite for another 2 h, 75% Epon-Aradite overnight, and 100% Epon-Aradite for a day with one change before being embedded in two permanox slides by flat-embedding procedure in a 60°C oven for 48 h. The polymerized tissue blocks were cut into ultrathin sections (80 nm), which were collected on 150 mesh grids. and examined under an FEI CM120 transmission electron microscope (equipped with a Gatan GIF100 image filter) operating at a beam energy of 120 keV. Images were acquired using a Gatan 1 k × 1 k cooled charge-coupled device (CCD) camera.

### Data analysis

Statistical analyses were performed using the Student’s t-test or two-way Anova using Graphpad Prism 5.0. Post-hoc Bonferroni’s correction was used for multiple comparisons. *p*-values<0.05 were considered statistically significant.

## Supporting Information

S1 Fig Acer3 knockdown nearly abolishes alkaline ceramidase activity in the lungs and liver. **A**. Alkaline ceramidase activity on NBD-C_12_-PHC in Acer3 knockout lung and liver tissues is drastically diminished. Total cellular membranes were isolated from lung or liver tissues of Acer3^+/+^ and Acer3^-/-^ mice at 6W of age and measured for alkaline ceramidase activity using NBD-C12-PHC as a substrate. **B.** Alkaline ceramidase activity on C_18:1_-ceramide in Acer3 knockout lung and liver tissues is drastically diminished. Data represent mean values ± SD, n = 3; ***p<0.001.
(TIF)

S2 Fig Acer3 knockdown does not affect locomotor activity at middle age. **A**-**E**. Open field tests: Acer3^+/+^ and Acer3^-/-^ mice at 6W, 8M, or 12M of age were placed in an open field and their open field activities were recorded for 5 min. Representative footprint pathways from one mouse in each group are illustrated in (A), and walking distance (B), velocity (C), corner latency (D), and rearing activity (E) quantified from all tested mice. The data in B, C, D, and E represent mean values ± SD, n = 6; *p<0.05, ***p<0.001.
(TIF)

S3 Fig Acer3 knockdown does not affect footprint pattern at middle age. **A** and **B**. Footprint tests: Representative footprint patterns (A) are shown from Acer3^+/+^ and Acer3^-/-^ mice at 6W, 8M, and 12M. The stride length, hindpaw step width, forepaw step width, and the extent of overlap between the forepaws and hindpaws (paw overlap) were quantified (B). The data in B represent mean values ± SD, n = 6.
(TIF)

S4 Fig Acer3 knockout does not affect muscle strength. Muscle strength of the forepaw (A) and hindpaw (B) in Acer3^+/+^ and Acer3^-/-^ mice at 4M, 6M, 8M or 12M of age was determined using a grip-strength meter. The data represent mean values ± SD, n = 4–9 mice per group.
(TIF)

S5 Fig Acer3 knockout does not alter basal levels of apoptosis in the lungs or liver. **A** and **B**. Western blot analysis: Representative images from Western blot analyses for the cleavage of caspase 3 in lung (A) or liver (B) tissues are shown. Lung and liver tissues were collected from 6-week-old Acer3^+/+^ or Acer3^-/-^ mice and the cleavage of caspase 3 was analyzed. **C**-**F**. TUNEL assays for apoptosis. Representative horizontal sections of lung and liver tissue from an Acer3^+/+^ and Acer3^-/-^ mouse was stained with a TUNEL kit and imaged under the microscope (C). TUNEL-positive cells (indicated with arrowheads) in bronchial epithelium (D) and alveoli (E) or liver (F) were subsequently quantified. The data in D–F represent mean values ± SD, n = 4; **p<0.01.
(TIF)

S6 Fig Acer3 knockout does not affect basal proliferation in the lungs or liver. **A**-**C**. Western blot analyses for PCNA. Liver or lung tissues from 6-week-old Acer3^+/+^ or Acer3^-/-^ were analyzed for PCNA expression using anti-PCNA antibody (A). Protein band density was determined by densitometry to estimate PCNA expression in lungs (B) and liver (C). **D**-**F**. Representative Ki67 staining of proliferating cells in the lungs or liver from Acer3^+/+^ and Acer3^-/-^ mice. After immunostaining with anti-Ki67 antibody, tissue sections were imaged under the microscope (D) and Ki67-positive cells (indicated by arrowheads) in the bronchial epithelium and alveoli of lungs or hepatocytes (indicated by arrowheads) and non-hepatocytes (indicated by arrows) in the liver were quantified (E and F). The data in B, C, E, and F represent mean values ± SD, n = 4 mice per group.
(TIF)

S7 Fig Acer3^+/-^ mice exhibit a normal profile of sphingolipids and motor coordination. **A** and **B**. Rotarod and hindlimb clasping tests on Acer3^+/-^ mice at 8M of age. Rotarod and hindlimb clasping tests were performed on Acer3^+/+^ and Acer3^+/-^ mice at 8M. **C**. Alkaline ceramidase activity on NBD-C_12_-PHC in brain of Acer3^+/-^. Total cellular membranes were isolated from brian tissues of Acer3^+/+^ and Acer3^+/-^ mice at 8M of age and measured for alkaline ceramidase activity using NBD-C12-PHC as a substrate. **D** and **E**. Ceramide levels of brain tissues of Acer3^+/-^ and Acer3^+/+^ mice at 8M. Cerebral (D) and cerebellar (E) tissues were collected from 8-month-old Acer3^+/+^ and Acer3^+/-^ mice and subjected to LC-MS/MS analyses for the levels of ceramides, C_18:1_-ceramide and total ceramide. The data represent mean values ± SD, n = 5 mice per group.
(TIF)

S1 Table Acer3 deficiency alters levels of ULCCs and their metabolites in the lungs and liver. Lung and liver tissues were collected from 6-week-old Acer3^+/+^ and Acer3^-/-^ mice and subjected to LC-MS/MS analysis for the levels of individual ceramides, SPH, S1P, monohexosylceramides, and sphingomyelins. Total levels of sphingolipids were the sum of individual species of the same class. The data represent mean values ± SD, n = 4; *p<0.05, **p<0.01, ***p<0.001, Acer3^+/+^ vs. Acer3^-/-^.
(TIF)

S1 Video Inverted wire hanging tests. The inverted wire hanging tests was performed to screen for any behavioral deficits in the Acer3^+/+^ and Acer3^-/-^ mice at 3M, 6M, 8M, and 10M.
(MP4)

S2 Video Rotarod tests. In a pair of representative 8-month-old Acer3^+/+^ and age-matched Acer3^-/-^ mouse, the video demonstrates walking patterns (including paw placement) on the rotarod.
(MP4)

S3 Video Beam walking tests. **A** and **B**. In a pair of representative 8-month-old Acer3^+/+^ and Acer3^-/-^ mouse, the video demonstrates walking behavior on a balance beam (10-mm).
(MP4)
