# Correction: Alkaline Ceramidase 3 Deficiency Results in Purkinje Cell Degeneration and Cerebellar Ataxia Due to Dyshomeostasis of Sphingolipids in the Brain

**DOI:** 10.1371/journal.pgen.1007190

**Published:** 2018-01-23

**Authors:** Kai Wang, Ruijuan Xu, Jennifer Schrandt, Prithvi Shah, Yong Z. Gong, Chet Preston, Louis Wang, Jae Kyo Yi, Chih-Li Lin, Wei Sun, Demetri D. Spyropoulos, Soyoung Rhee, Mingsong Li, Jie Zhou, Shaoyu Ge, Guofeng Zhang, Ashley J. Snider, Yusuf A. Hannun, Lina M. Obeid, Cungui Mao

There are errors in Figs 4A and 9A and S2A, and in the primer sequences listed for *Acer2* and *Acer3* in the ‘RNA extraction and qPCR’ section of the Materials and Methods. The authors apologize for the errors and provide further details and corrections below.

In Fig 4A, an incorrect pair of primers was used to amplify the whole open reading frame of the *Acer3* gene. The correct primer pair is 5'-ATGGCTCCGGCTGTGGACC-3'/5'-TCAGTGCTTCCTCTGAGGTTCAAAC-3'. The authors have redone the experiment with the correct primer pair, and have provided a corrected Fig 4. In Fig 9A, the histology panel of the Acer3 knockout mouse (Acer3-/-) for the 6W timepoint is duplicated. The histology panel has been corrected in the new Fig 9. In S2A Fig, the wild-type (Acer3+/+) panel is duplicated for the 12M timepoints. The correct Acer3 knockout (Acer3-/-) panel is provided in the revised S2 Fig.

**Fig 4:**
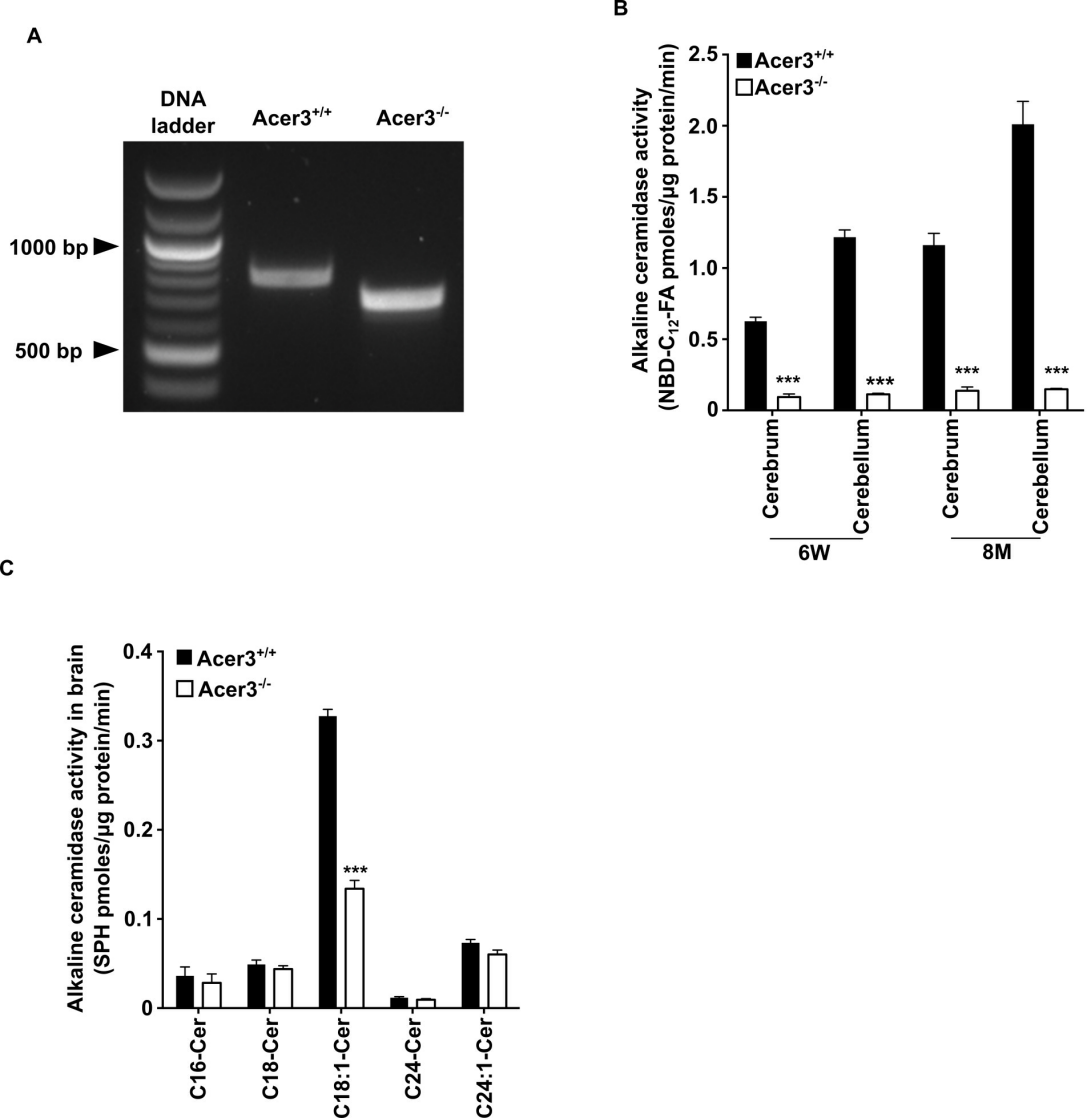
Acer3 knockout decreases alkaline ceramidase activity on ULCC in the brain. A. The transcription of a truncated coding sequence in a representative Acer3 knockout mouse. RNAs were isolated from the brains of Acer3^+/+^ or Acer3^-/-^ mice and subjected to RT-PCR using a pair of primers encompassing the start codon and stop codon, respectively, of the Acer3 gene. Note that the Acer3^-/-^ mouse has a smaller ORF of the Acer3 gene than an age-matched Acer3^+/+^ mouse. **B**. Reduction of alkaline ceramidase activity on NBD-C_12_-PHC in Acer3^-/-^ mice. Note that Acer3^-/-^ mice at either 6W or 8M of age show significant declines in ceramidase activity in both the cerebellar and cerebral brains compared to their WT littermates. **C**. Reduction of alkaline ceramidase activity on C_18:1_-ceramide in the whole brains of Acer3 knockout mice. Note that the brain alkaline ceramidase activity on this ceramide was substantially decreased in Acer3^-/-^ mice compared to Acer3^+/+^ mice. Image in A represents result from 3 pairs of mice. Data in B and C represent mean values ± SD, n = 3.

**Fig 9:**
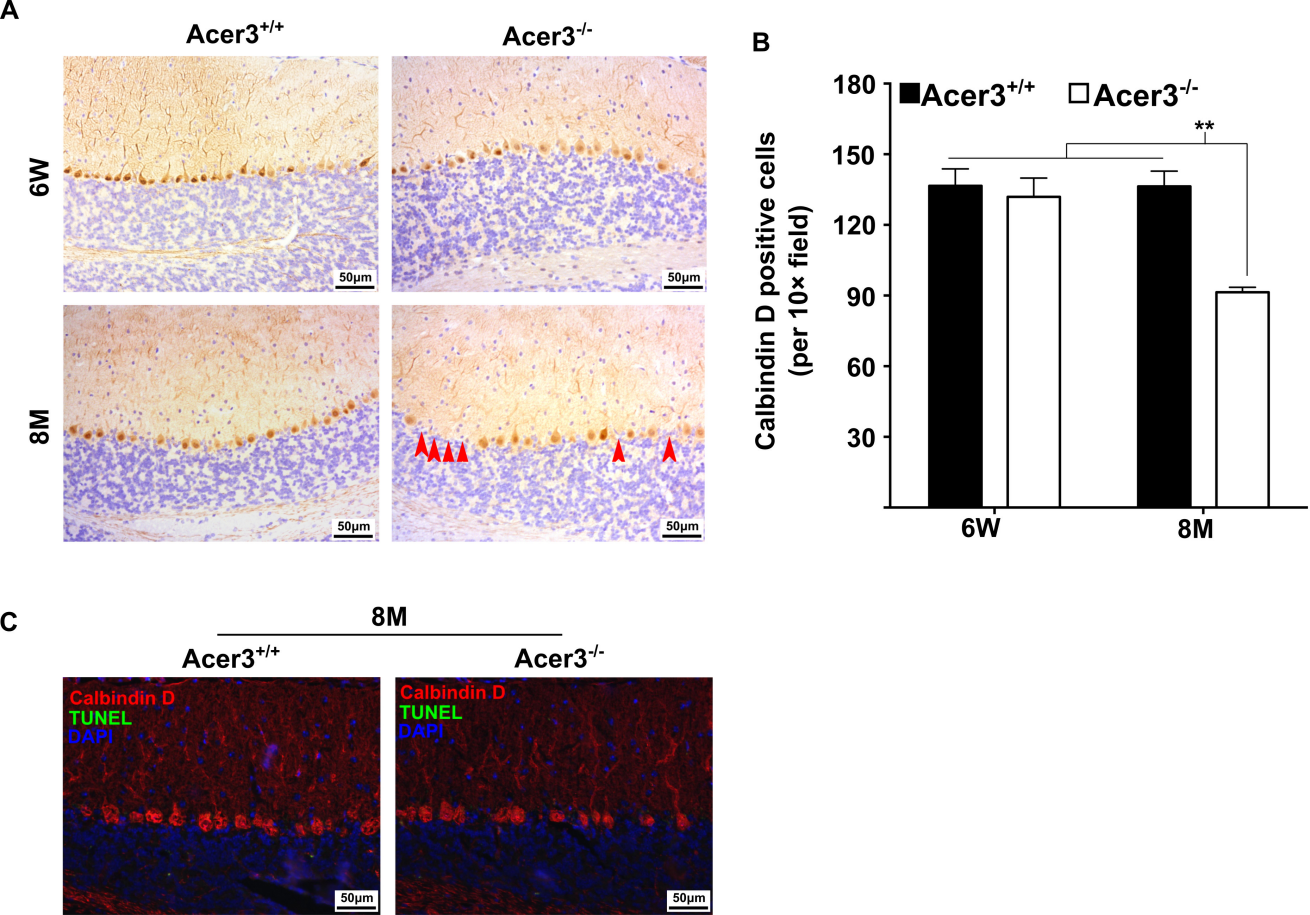
Acer3 knockout induces premature degeneration of PCs. **A** and **B**. PC loss in Acer3 knockout mice at 8M of age. Immunostaining of cerebellar sagittal sections with antibody against calbindin D-28K, a PC marker (A). Red arrowheads indicate the regions where PCs were lost. Quantification of PCs (B). Images in A are the results from a representative mouse in each group. **C**. TUNEL assays for apoptosis in the cerebellum from Acer3^+/+^ and Acer3^-/-^ mice. The cerebellar sections of Acer3^+/+^and Acer3^-/-^ mice at 8M of age were co-stained with the TUNEL assay reagent (green fluorescence) and anti-calbindin D28K antibody (red fluorescence). The images in A and C are the results from a representative mouse in each group. The data in B represent mean values ± SD, n = 4.

The primer sequences listed for *Acer2* and *Acer3* in the ‘RNA extraction and qPCR’ section of the Materials and Methods are duplicated. The correct primer sequences for *Acer3* are 5'-GATTCACTGAGGAACTTTCG-3'/5'-AGAGAAACTTCACTTTTGGC-3'. The primer sequences for *Acer2* are correct.

The underlying spreadsheet data for the graphs and bar charts in the figures is not included in the published article, and is available as S1 Dataset.

## Supporting information

S2 Fig Acer3 knockdown does not affect locomotor activity at middle age. **A**-**E**. Open field tests: Acer3^+/+^ and Acer3^-/-^ mice at 6W, 8M, or 12M of age were placed in an open field and their open field activities were recorded for 5 min. Representative footprint pathways from one mouse in each group are illustrated in (A), and walking distance (B), velocity (C), corner latency (D), and rearing activity (E) quantified from all tested mice. The data in B, C, D, and E represent mean values ± SD, n = 6; *p<0.05, ***p<0.001.
(TIF)

S1 Dataset Underlying data for Figs 1 & 3–10, S1 –S7 Figs and S1 Table. (XLSX)
