# Supplementary figures and images for: Alkaline Ceramidase 3 Deficiency Results in Purkinje Cell Degeneration and Cerebellar Ataxia Due to Dyshomeostasis of Sphingolipids in the Brain
Source: PLoS Genet. 2015 Oct 16;11(10):e1005591. doi: 10.1371/journal.pgen.1005591 (PMC4608763; doi:10.1371/journal.pgen.1005591)

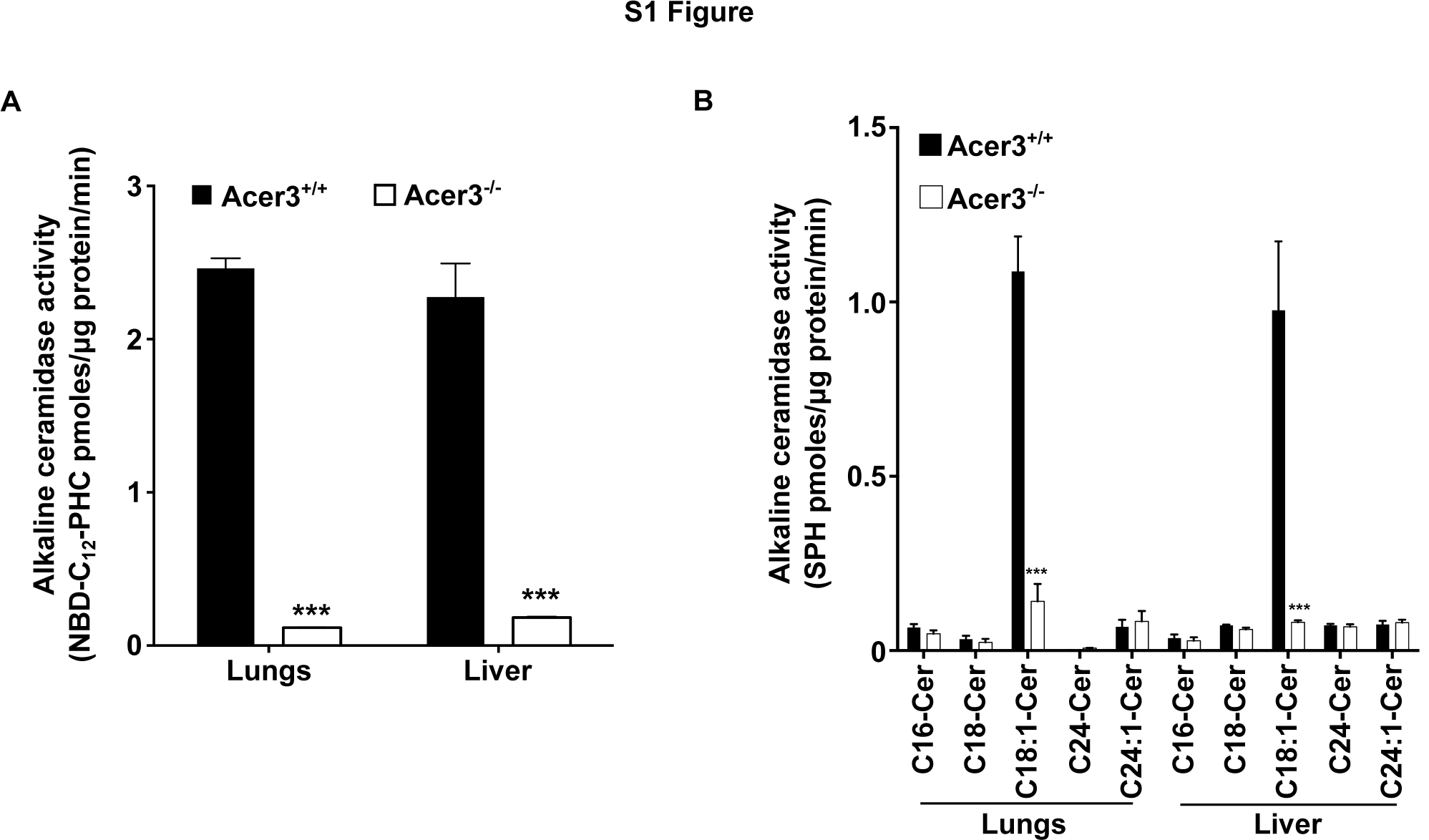

Supplement: S1 Fig — A. Alkaline ceramidase activity on NBD-C12-PHC in Acer3 knockout lung and liver tissues is drastically diminished. Total cellular membranes were isolated from lung or liver tissues of Acer3+/+ and Acer3-/- mice at 6W of age and measured for alkaline ceramidase activity using NBD-C12-PHC as a substrate. B. Alkaline ceramidase activity on C18:1-ceramide in Acer3 knockout lung and liver tissues is drastically diminished. Data represent mean values ± SD, n = 3; ***p<0.001. (TIF) [file pgen.1005591.s001.tif]

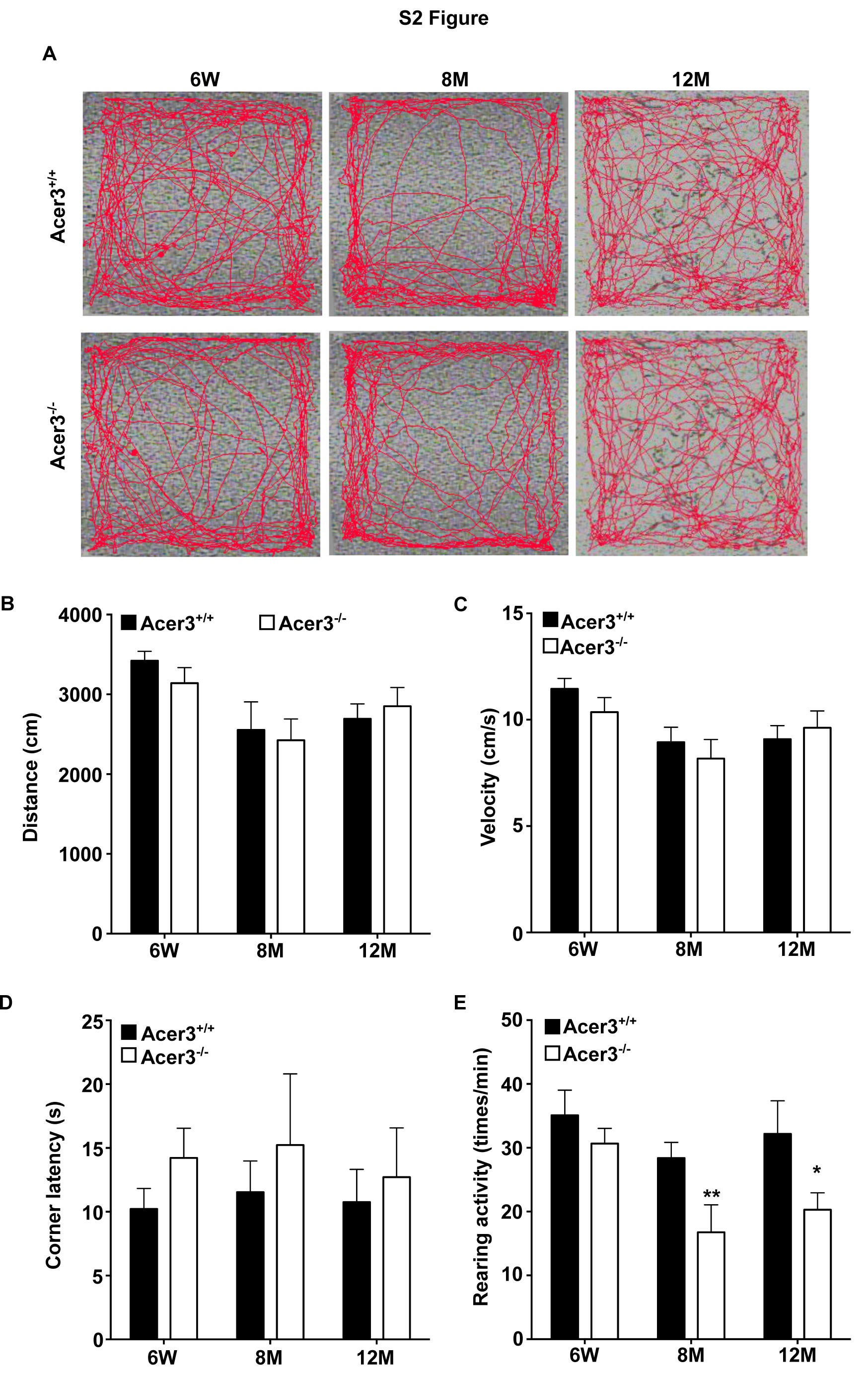

Supplement: S2 Fig — A-E. Open field tests: Acer3+/+ and Acer3-/- mice at 6W, 8M, or 12M of age were placed in an open field and their open field activities were recorded for 5 min. Representative footprint pathways from one mouse in each group are illustrated in (A), and walking distance (B), velocity (C), corner latency (D), and rearing activity (E) quantified from all tested mice. The data in B, C, D, and E represent mean values ± SD, n = 6; *p<0.05, ***p<0.001. (TIF) [file pgen.1005591.s002.tif]

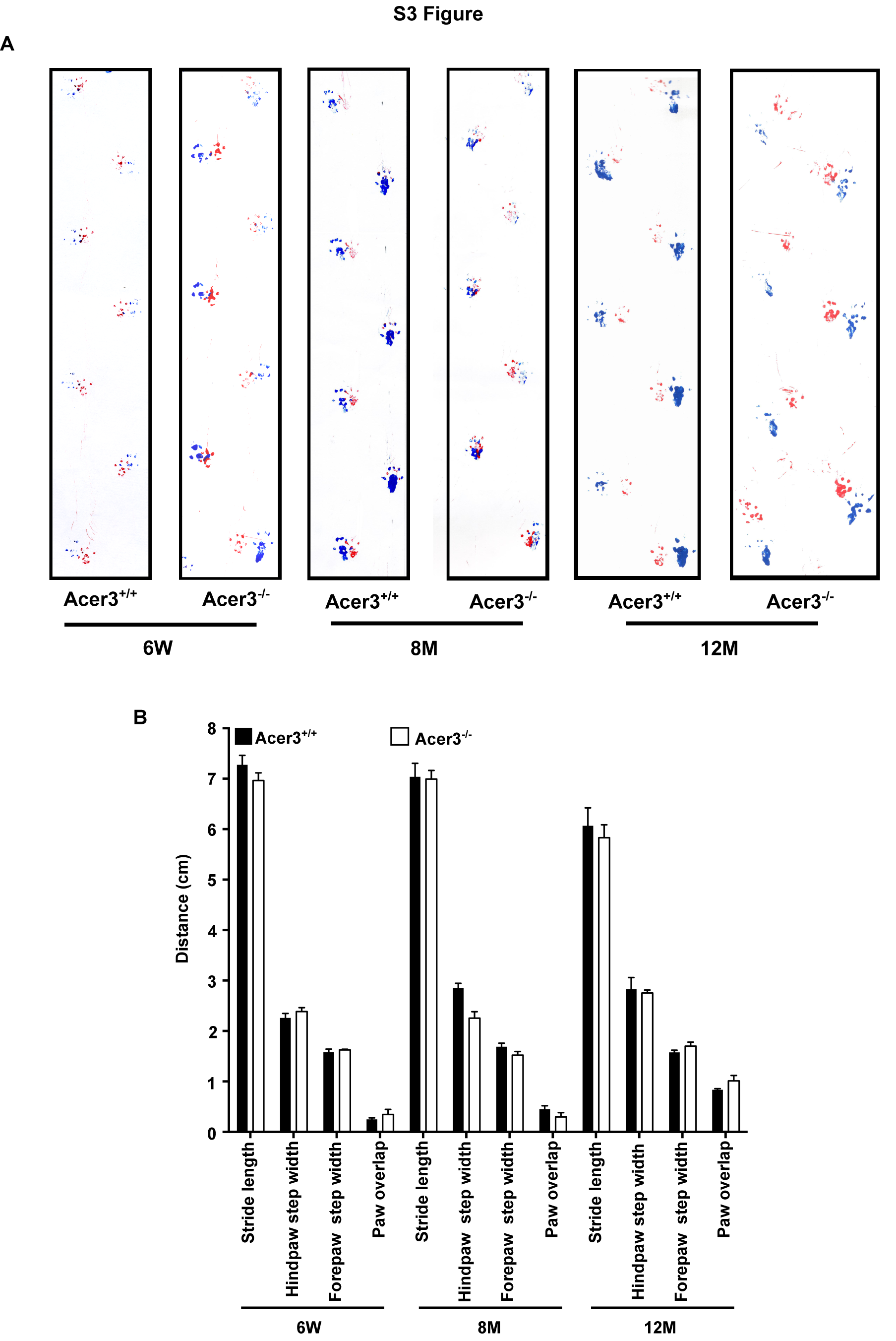

Supplement: S3 Fig — A and B. Footprint tests: Representative footprint patterns (A) are shown from Acer3+/+ and Acer3-/- mice at 6W, 8M, and 12M. The stride length, hindpaw step width, forepaw step width, and the extent of overlap between the forepaws and hindpaws (paw overlap) were quantified (B). The data in B represent mean values ± SD, n = 6. (TIF) [file pgen.1005591.s003.tif]

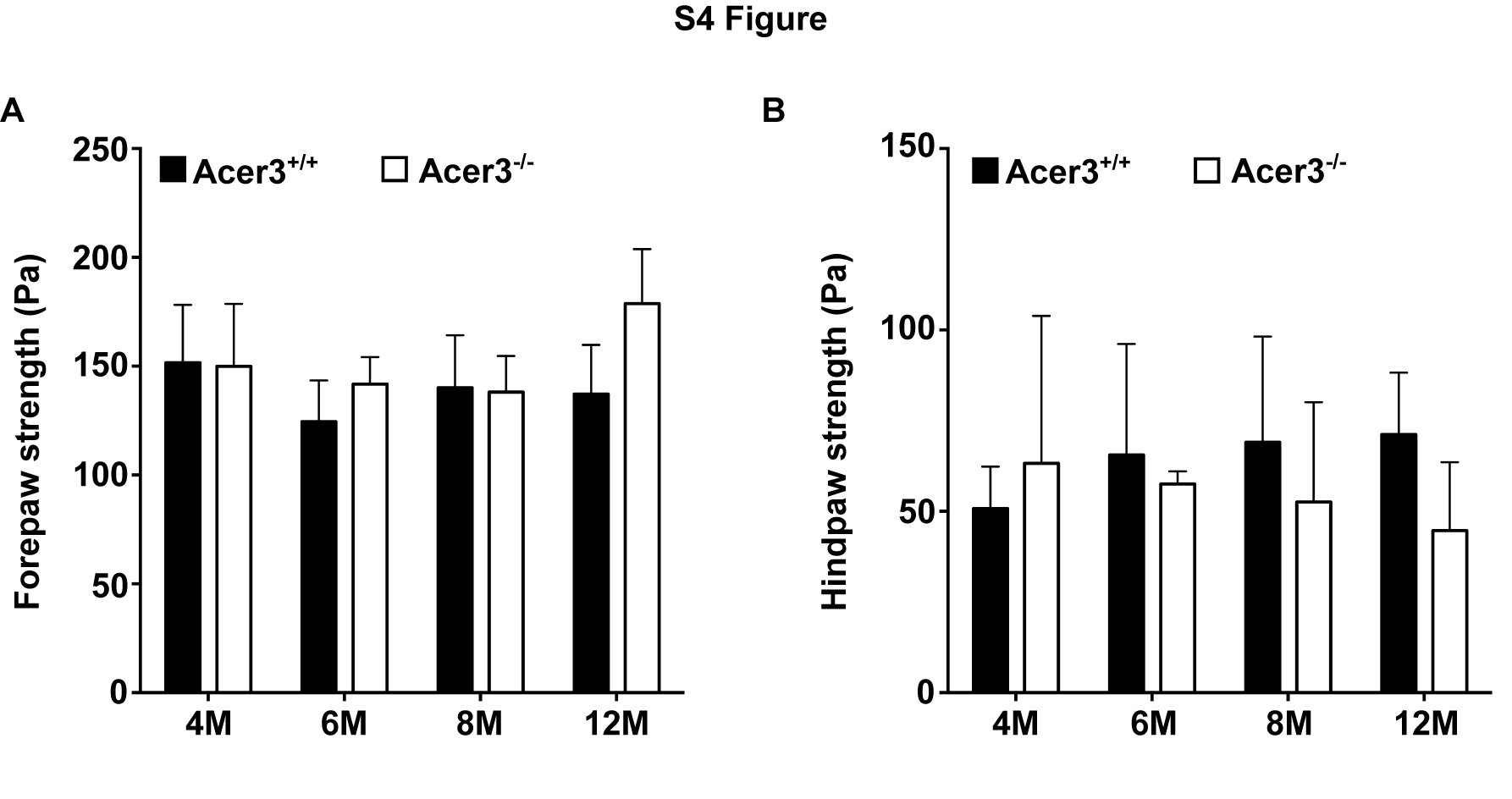

Supplement: S4 Fig — Muscle strength of the forepaw (A) and hindpaw (B) in Acer3+/+ and Acer3-/- mice at 4M, 6M, 8M or 12M of age was determined using a grip-strength meter. The data represent mean values ± SD, n = 4–9 mice per group. (TIF) [file pgen.1005591.s004.tif]

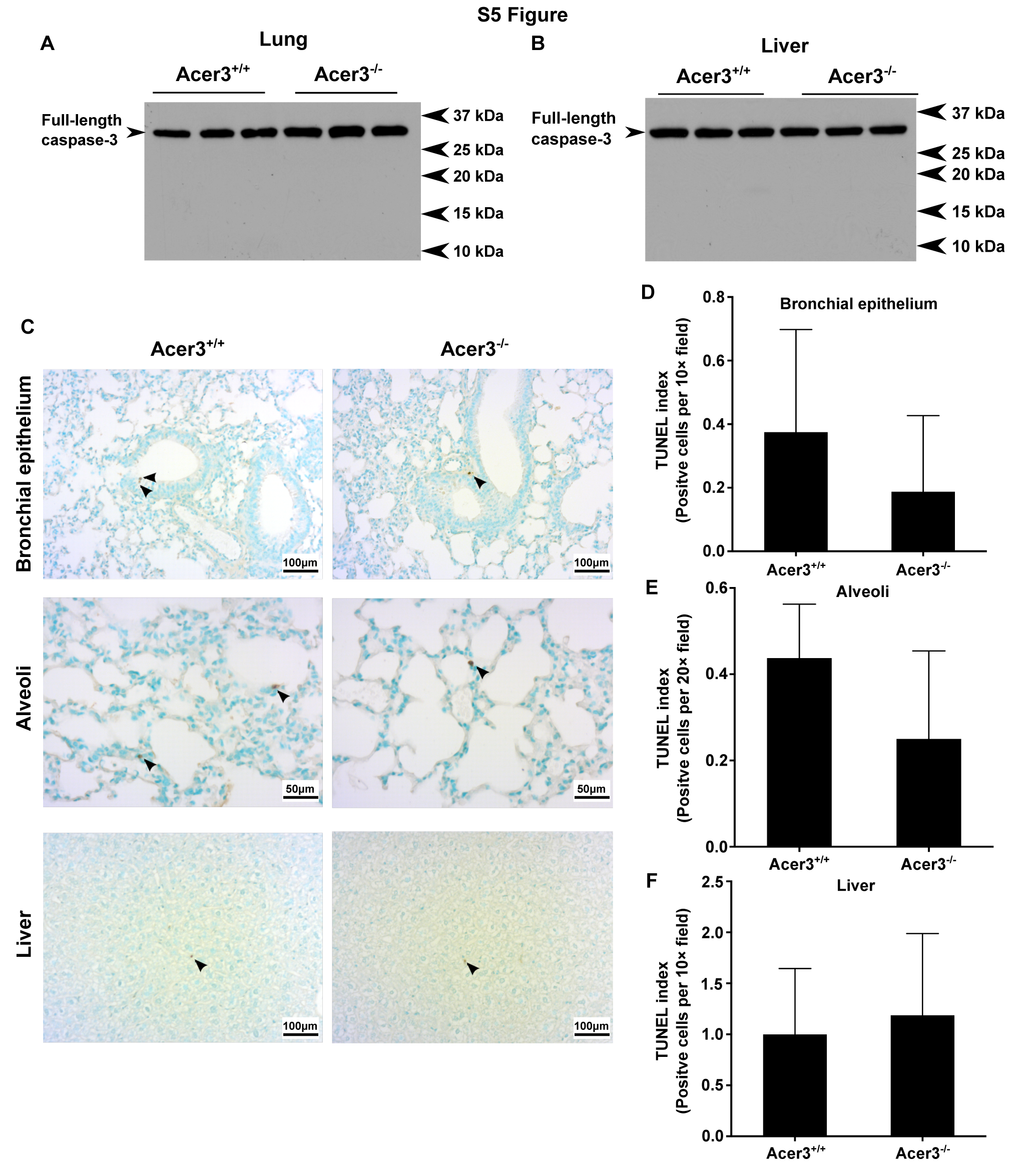

Supplement: S5 Fig — A and B. Western blot analysis: Representative images from Western blot analyses for the cleavage of caspase 3 in lung (A) or liver (B) tissues are shown. Lung and liver tissues were collected from 6-week-old Acer3+/+ or Acer3-/- mice and the cleavage of caspase 3 was analyzed. C-F. TUNEL assays for apoptosis. Representative horizontal sections of lung and liver tissue from an Acer3+/+ and Acer3-/- mouse was stained with a TUNEL kit and imaged under the microscope (C). TUNEL-positive cells (indicated with arrowheads) in bronchial epithelium (D) and alveoli (E) or liver (F) were subsequently quantified. The data in D–F represent mean values ± SD, n = 4; **p<0.01. (TIF) [file pgen.1005591.s005.tif]

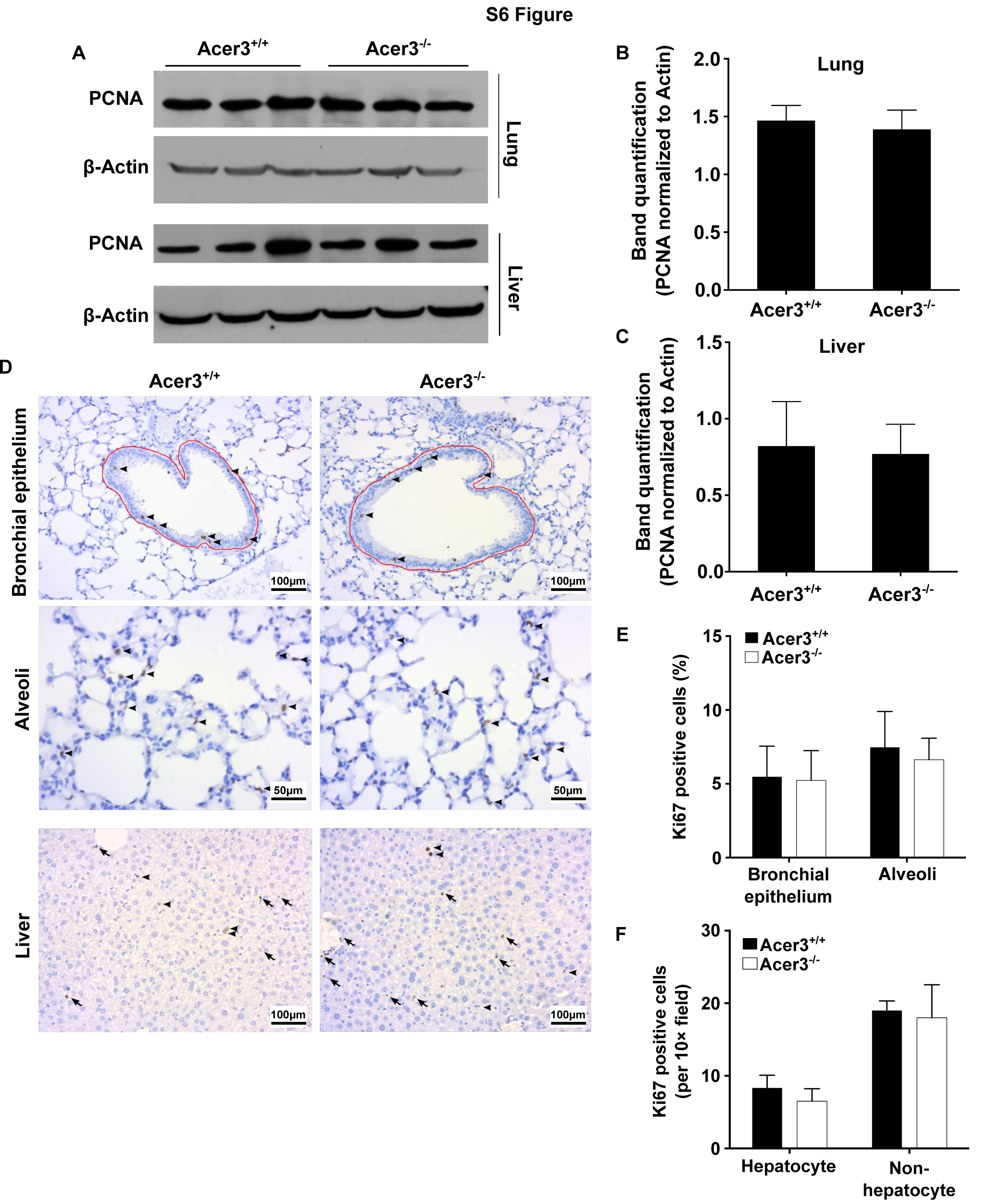

Supplement: S6 Fig — A-C. Western blot analyses for PCNA. Liver or lung tissues from 6-week-old Acer3+/+ or Acer3-/- were analyzed for PCNA expression using anti-PCNA antibody (A). Protein band density was determined by densitometry to estimate PCNA expression in lungs (B) and liver (C). D-F. Representative Ki67 staining of proliferating cells in the lungs or liver from Acer3+/+ and Acer3-/- mice. After immunostaining with anti-Ki67 antibody, tissue sections were imaged under the microscope (D) and Ki67-positive cells (indicated by arrowheads) in the bronchial epithelium and alveoli of lungs or hepatocytes (indicated by arrowheads) and non-hepatocytes (indicated by arrows) in the liver were quantified (E and F). The data in B, C, E, and F represent mean values ± SD, n = 4 mice per group. (TIF) [file pgen.1005591.s006.tif]

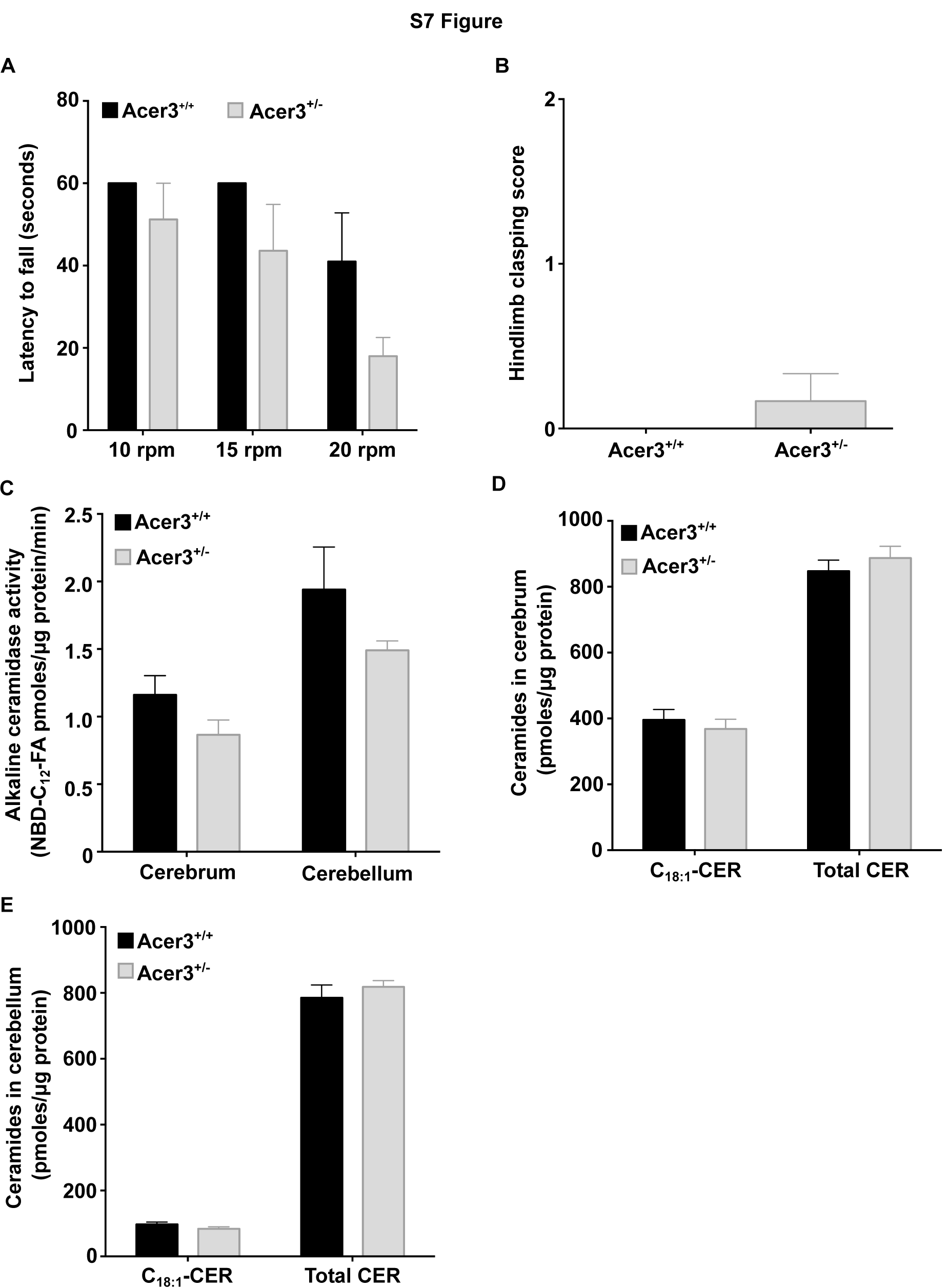

Supplement: S7 Fig — A and B. Rotarod and hindlimb clasping tests on Acer3+/- mice at 8M of age. Rotarod and hindlimb clasping tests were performed on Acer3+/+ and Acer3+/- mice at 8M. C. Alkaline ceramidase activity on NBD-C12-PHC in brain of Acer3+/-. Total cellular membranes were isolated from brian tissues of Acer3+/+ and Acer3+/- mice at 8M of age and measured for alkaline ceramidase activity using NBD-C12-PHC as a substrate. D and E. Ceramide levels of brain tissues of Acer3+/- and Acer3+/+ mice at 8M. Cerebral (D) and cerebellar (E) tissues were collected from 8-month-old Acer3+/+ and Acer3+/- mice and subjected to LC-MS/MS analyses for the levels of ceramides, C18:1-ceramide and total ceramide. The data represent mean values ± SD, n = 5 mice per group. (TIF) [file pgen.1005591.s007.tif]

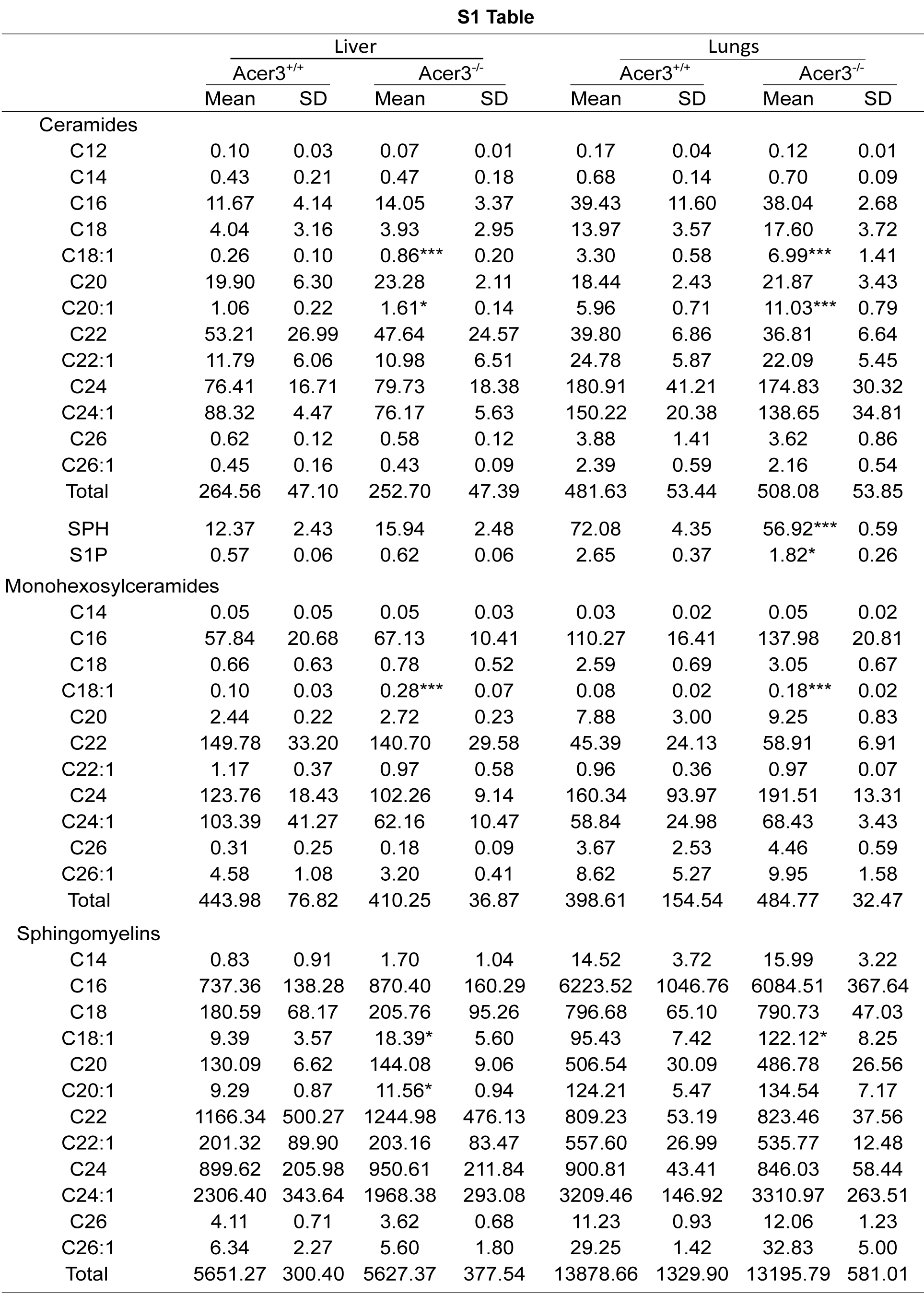

Supplement: S1 Table — Lung and liver tissues were collected from 6-week-old Acer3+/+ and Acer3-/- mice and subjected to LC-MS/MS analysis for the levels of individual ceramides, SPH, S1P, monohexosylceramides, and sphingomyelins. Total levels of sphingolipids were the sum of individual species of the same class. The data represent mean values ± SD, n = 4; *p<0.05, **p<0.01, ***p<0.001, Acer3+/+ vs. Acer3-/-. (TIF) [file pgen.1005591.s008.tif]
